# Environmental Dissemination of Antimicrobial Resistance: A Resistome-Based Comparison of Hospital and Community Wastewater Sources

**DOI:** 10.3390/antibiotics15010099

**Published:** 2026-01-19

**Authors:** Taito Kitano, Nobuaki Matsunaga, Takayuki Akiyama, Takashi Azuma, Naoki Fujii, Ai Tsukada, Hiromi Hibino, Makoto Kuroda, Norio Ohmagari

**Affiliations:** 1 AMR Clinical Reference Center, National Center for Global Health and Medicine, Japan Institute for Health Security, Tokyo 162-8655, Japan; kitano.t@jihs.go.jp (T.K.); akiyama.t@jihs.go.jp (T.A.); fujii.na@jihs.go.jp (N.F.); tsukada.ai@jihs.go.jp (A.T.); ohmagari.n@jihs.go.jp (N.O.); 2 Disease Control and Prevention Center, National Center for Global Health and Medicine, Japan Institute for Health Security, Tokyo 162-8655, Japan; 3 Large-Scale Data Archiving and Processing Section, Institute of Economic Research, Hitotsubashi University, Tokyo 186-0004, Japan; 4 Department of Pharmacy, Osaka Medical and Pharmaceutical University, Takatsuki 569-1094, Japan; takashi.azuma@ompu.ac.jp; 5 Center for Infectious Disease Education and Research (CiDER), Osaka University, Suita 565-0871, Japan; 6 Research and Development Coordination Office, National Center for Global Health and Medicine, Japan Institute for Health Security, Tokyo 162-8655, Japan; hibino.h@jihs.go.jp; 7 Pathogen Genomics Center, National Institute of Infectious Diseases, Tokyo 162-0052, Japan; kuroda@kumamoto-hsu.ac.jp; 8 Department of Medical Technology, Faculty of Health Sciences, Kumamoto Health Science University, 325 Izumi-machi, Kita-ku, Kumamoto 861-5598, Japan

**Keywords:** antimicrobial resistance (AMR), wastewater, hospital, community, antimicrobial stewardship

## Abstract

**Background/Objectives:** Comparative analysis of antimicrobial resistomes in hospital and community wastewater can provide valuable insights into the diversity and distribution of antimicrobial resistance genes (ARGs), contributing to the advancement of the One Health approach. This study aimed to characterize and compare the resistome profiles of wastewater sources from a hospital and community. **Methods:** Longitudinal metagenomic analysis was conducted on wastewater samples collected from the National Center for Global Health and Medicine (hospital) and a shopping mall (community) in Tokyo, Japan, between December 2019 and September 2023. ARG abundance was quantified using reads per kilobase per million mapped reads (RPKM) values, and comparative analyses were performed to identify the significantly enriched ARGs in the two sources. **Results:** A total of 46 monthly wastewater samples from the hospital yielded 825 unique ARGs, with a mean RPKM of 2.5 across all detected genes. In contrast, 333 ARGs were identified in the three shopping mall wastewater samples, with a mean RPKM of 2.1. Among the ARGs significantly enriched in the hospital samples, 23, including genes conferring resistance to aminoglycosides (nine groups) and *β*-lactam antibiotics (eight groups), exhibited significantly high RPKM values. No ARGs were found to be significantly enriched in the community wastewater samples. **Conclusions:** This study highlights the higher diversity and abundance of ARGs, particularly those conferring resistance to aminoglycosides and *β*-lactam antibiotics including carbapenems, in hospital wastewater than in community wastewater. These findings underscore the importance of continuous resistome monitoring of hospital wastewater as part of the integrated One Health surveillance strategy.

## 1. Introduction

Antimicrobial resistance (AMR) is associated with a substantial number of deaths worldwide and considered one of the most pressing global public health threats [1]. Antimicrobial use, including the use of broad-spectrum agents, is a major factor leading to the emergence of antimicrobial-resistant organisms (AROs) and AMR genes (ARGs). The One Health framework, which integrates human, animal, and environmental health, is essential for comprehensively addressing the emergence and dissemination of AMR [2].

Wastewater systems are recognized as critical reservoirs of AROs and ARGs and serve as ecological niches facilitating the extensive horizontal gene transfer of resistance elements [3,4]. In these systems, various selection pressures, including residual antimicrobials, heavy metals, and disinfectants, promote both the selection and coselection of resistance genes [5]. Monitoring ARG dynamics in wastewater is critical for the early detection of emerging resistance threats and understanding environmental transmission routes. Environmental bacteria can harbor ARGs, which may be horizontally transferred to pathogenic strains capable of colonizing human hosts and causing infections [6,7]. These facilities contribute significantly to the environmental load of ARGs. Wastewater-based epidemiology (WBE) has emerged as a viable and promising approach to monitor AMR at the community level. Wastewater represents an aggregated matrix of microbial and chemical signatures, offering an integrative proxy for assessing the community-level AMR burden.

Elucidating the relationship between residual antimicrobials and the resistome in wastewater is particularly critical, as it can inform targeted surveillance strategies and facilitate the effective mitigation of environmental AMR risks. Healthcare facilities, especially tertiary and quaternary care hospitals, are major sources of AMR dissemination due to the frequent use of broad-spectrum antimicrobials and presence of patients infected with or colonized by multidrug-resistant organisms. Therefore, these facilities contribute significantly to the burden of AMR and ARGs in wastewater.

A systematic review indicated the higher prevalence of AMR determinants (e.g., ARGs) in hospital wastewater than in community sources [8]. However, substantial heterogeneity exists among the studies in terms of the specific AMR traits evaluated, possibly reflecting the regional differences in resistance epidemiology and antimicrobial usage patterns. Comprehensive resistome analyses, including metagenomic sequencing, have been increasingly used to understand this complexity [9]. Unlike targeted ARG assays, these approaches enable the detection of both clinically relevant and previously uncharacterized resistance genes [10], thereby providing a broader and more informative perspective on AMR emergence, including the underlying resistance determinants and their potential reservoirs.

Despite their recognized roles in AMR propagation, the interactions between resistome profiles and residual antimicrobial concentrations in these settings remain poorly understood [11]. To investigate this, we focused on the National Center for Global Health and Medicine (NCGM), a tertiary care hospital in central Tokyo affiliated with the Japan Institute for Health Security. This facility and its adjacent residential area provided an ideal setting for the comparative analysis of hospital and municipal wastewater resistomes.

The objective of this study was to characterize the antimicrobial resistomes and quantify the residual antimicrobial concentrations in the wastewater samples from a tertiary care hospital and its adjacent community. Our integrated approach aimed to advance our understanding of AMR dissemination in the environment and guide evidence-based One Health strategies for surveillance and mitigation.

## 2. Results

### 2.1. ARGs in the Wastewater Samples from the Hospital and Shopping Mall

From the 46 hospital wastewater samples, 825 ARGs with a mean RPKM value of 2.5 were detected among all 863 detected ARGs. On the other hand, 333 ARGs with a 2.1 mean RPKM value among all genes were detected in the three shopping mall wastewater samples. The list of detected ARGs and their mean RPKM values in the hospital and shopping mall samples are presented in Table S1.

**Figure 1:**
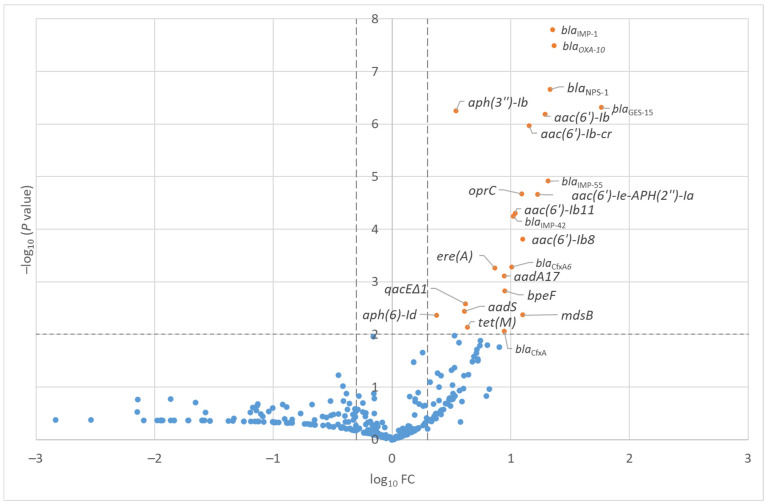
Volcano plot of the ratios of the mean reads per kilobase per million mapped reads (RPKM) values between the hospital and community wastewater samples from December 2019 to September 2023. Significantly higher RPKM (−log_10_ *p*-value > 2.0 and |log_10_ FC| > 0.3) values in the hospital samples were highlighted with orange color with ARG name, while blue color indicated non-significance.

Of the 46 hospital wastewater samples (Table S2), there was no increase in the mean RPKM values of all the investigated ARGs by antimicrobial class over time (Figure S1; Table S3). Figure 1 compares the RPKM values between the hospital and shopping mall samples. Significantly higher RPKM (−log_10_ *p*-value > 2.0 and |log_10_ FC| > 0.3) values in the hospital samples were observed for 23 ARGs.

The full list of ARGs with significantly higher RPKM in the hospital samples compared with the shopping mall samples is presented in Table 1. Of the 23 ARGs with significantly high RPKM values in the hospital samples, nine ARG groups (*aph(3″)-Ib*, *aac(6′)-Ib′*, *aac(6′)-Ib-cr*, *aac(6′)-Ie-aph(2″)-Ia*, *aac(6′)-Ib11*, *aac(6′)-Ib8*, *aadA17*, *aadS*, and *aph(6)-Id*) were associated with aminoglycosides, eight ARG groups (*bla*_IMP-1_, *bla*_OXA-10_, *bla*_NPS-1_, *bla*_GES-15_, *bla*_IMP-55_, *bla*_IMP-42_, *bla*_CfxA6_, and *bla*_CfxA_ groups) were associated with *β*-lactam antibiotics, three groups (*oprC*, *bpeF*, and *mdsB*) were associated with multidrug resistance, one group (*ere(A)*) was associated with macrolides, one group (*tet(*M*)*) was associated with tetracyclines, and one group (*qacEΔ1*) was associated with disinfectant resistance. On the other hand, there were no ARGs with significantly high RPKM values in the shopping mall samples.

Clinically important ARGs associated with carbapenemase-producing *Enterobacteriaceae* (CPE), other than the *bla*_IMP_, *bla*_NDM_, and *bla*_KPC_ groups, were not detected in any samples from the hospital and shopping mall wastewater systems. *bla*_VIM-1_ group was detected with mean RPKM values of 2.2 and 0.6 in the hospital and shopping mall samples, respectively (*p* = 0.061). None of the *bla*_CTX-M_ groups showed a significant difference in the RPKM value between the hospital and shopping mall samples.

### 2.2. Antimicrobials in the Wastewater Samples from the Hospital and Shopping Mall

A wide array of antimicrobials was detected in the wastewater samples, with concentrations ranging from 45 ng/L to 27 µg/L. Comprehensive quantitative data for each antimicrobial compound across different effluent types have been reported in our previous study [12]. Notably, clarithromycin was detected in 83% of hospital wastewater and in 100% of shopping mall wastewater samples. Levofloxacin was detected in both types of samples 100% of the time. The average detection concentrations were 2.3 µg/L and 12 µg/L for clarithromycin and levofloxacin, respectively, in hospital wastewater, and 202 ng/L and 68 ng/L in shopping mall wastewater. In the present investigation, we focused specifically on clarithromycin and levofloxacin, two compounds consistently detected in both hospital and shopping mall wastewater, and analyzed their concentration ratios to explore their sector-specific usage patterns.

Temporal variations in the hospital-to-shopping mall concentration ratios of these two antibiotics are summarized in Table S4. For clarithromycin, log_10_-transformed ratio ranged from −0.5 to 1.9, with a mean value of 0.6 ± 0.7, corresponding to an approximate four-fold elevation in the hospital effluent. Notable peaks were observed in December 2021 (1.9 log_10_) and November 2022 (1.1 log_10_), suggesting episodic increases in clinical usage or unregulated discharge events.

Levofloxacin exhibited markedly higher and more consistent ratios, ranging from 0.8 to 2.9 log_10_, with a mean of 1.9 ± 0.6 (equivalent to approximately 77-fold higher concentrations) in hospital wastewater. Elevated ratios were observed several times, in December and February 2021 (2.7 and 2.5 log_10_, respectively), October and November 2022 (2.8 and 2.9 log_10_, respectively) and April and May 2023 (2.7 and 2.5 log_10_, respectively), potentially indicating periods of intensified hospital administration or direct disposal.

**Figure 2:**
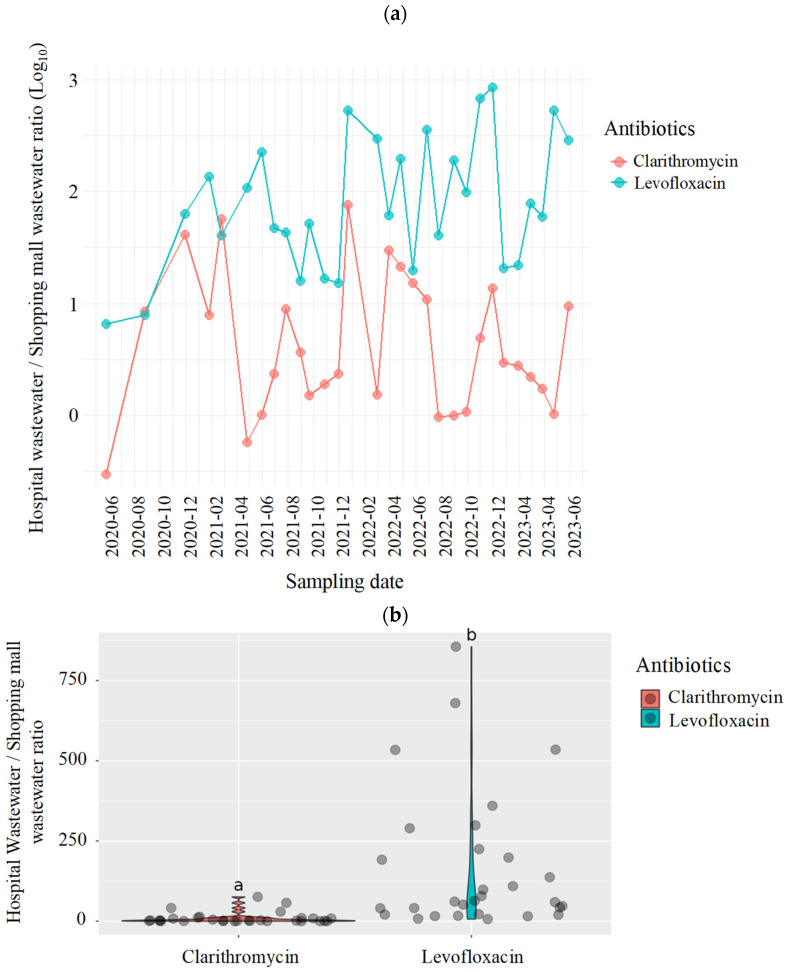
(**a**) Temporal variations in the concentration ratios and (**b**) distribution of the concentration ratios of clarithromycin and levofloxacin from June 2020 to June 2023. Different characters within the violin plot (a and b) indicate significant differences between groups. (*p* < 0.05).

Figure 2a shows the temporal trends in the log_10_ concentration ratios of both compounds from June 2020 to June 2023. Levofloxacin consistently maintained elevated values throughout the three-year monitoring period, generally ranging between 1.0 and 3.0 log_10_. In contrast, clarithromycin showed greater variability, with log_10_ ratios frequently falling below 1.0 and occasionally approaching or dropping below 0.0, particularly after 2021.

Distribution of the log_10_ concentration ratios of clarithromycin and levofloxacin (Figure 2b) and probability density functions of the log_10_ concentration ratios are presented in Figure S2. There were distinct variations in the log_10_ concentration ratios of clarithromycin and levofloxacin. The distribution of the density functions of the log_10_ concentration ratios of levofloxacin was unimodal, right-skewed, and centered around 1.5 log_10_, consistent with its persistent enrichment in the hospital effluent. In comparison, clarithromycin density was broader and more symmetrical, centered near 0.5 log_10_, reflecting the greater variability in its usage intensity and source contributions across sampling periods.

## 3. Discussion

This study evaluated and compared the antibiotic resistomes of wastewater from the hospital and shopping mall. Although the RPKM values of the 23 ARGs were significantly higher in the hospital samples than in the shopping mall samples, no ARGs had significantly high RPKM values in the shopping mall. Multiple factors, including patient characteristics and residual antibiotic concentrations in wastewater systems, can contribute to the higher abundance of ARGs in hospital wastewater systems than in communities [13,14,15].

In our study, we did not find any significant association between time and the number of ARGs associated with *β*-lactam antibiotics. Although the lack of temporal association with the number of ARGs warrants the comparison of the RPKM values for hospital and shopping mall samples across our study period, some studies have demonstrated the seasonality and temporal trends in the number of ARGs in hospital wastewater systems [16,17].

Our study found a higher abundance of various aminoglycoside resistance-associated ARGs in the hospital wastewater samples than in the shopping mall samples. Similar abundance of aminoglycoside resistance-associated ARGs in the wastewater systems of advanced hospitals has been reported by some studies worldwide [17,18,19,20].

RPKM values for the *bla*_IMP-1_, *bla*_IMP-42_, and *bla*_IMP-55_ groups were significantly higher in the hospital samples than in shopping mall samples in our study. In Japan, *bla*_IMP-1_ has been reported as one of the most common ARGs associated with CPE [21]. Although infections with carbapenem-resistant *Enterobacteriaceae* (CRE) are not very frequent in Japan (rate of carbapenem resistance was up to 0.6% across different species of *Enterobacteriaceae* between 2016 and 2022 based on nationwide Japanese data) [22], the potentially enormous negative impacts of CRE on morbidity and mortality have been recognized in the event of increased infections with CRE in the near future. The statistically significant difference in *bla*_IMP-1_ prevalence between the hospital and community samples observed in our study suggests that *bla*_IMP-1_ is not currently circulating in communities; however, advanced medical centers with many high-risk patients for CPE colonization and infection may harbor *bla*_IMP-1_ in their wastewater systems. This underscores the need for hospital wastewater surveillance of clinically important ARGs, irrespective of their history of detection among patients in every hospital. *bla*_KPC_ and *bla*_NDM_ groups were not detected in the hospital and shopping mall samples. The vast majority of clinical cases of CRE infection are associated with IMP, and only a few cases of CRE infection with KPC, NDM, OXA, or VIP have been clinically reported in Japan [23]. The lack of detection of the *bla*_KPC_ and *bla*_NDM_ groups in our study indicates the key role of hospital wastewater surveillance for the early detection of ARGs not yet circulating in the nation but possibly imported from other nations.

There were no statistically significant differences in the *bla*_CTX-M_ groups, which are the most frequently detected ARGs in extended spectrum *β*-lactamase (ESBL)-producing *Enterobacteriaceae* in Japan, between the hospital and shopping mall samples in our study. For example, the mean RPKM value of the *bla*_CTX-M-102_ group, including *bla*_CTX-M-27_, one of the most common ARGs associated with ESBL-producing *Enterobacteriaceae* detected in some Japanese surveillance reports [24], was 0.09 for the hospital samples and 0.31 for the shopping mall samples (*p* = 0.560). Third-generation cephalosporin resistance is common in Japan. According to the national surveillance data, third-generation cephalosporin resistance rate was 26.8% in *Escherichia coli* isolated from hospitalized patients in 2022 [25]. The results of our study indicate that some *bla*_CTX-M_ groups are ubiquitous in both the hospital and community wastewater systems in Japan.

The consistently elevated concentrations and detection frequencies of antimicrobials in hospital wastewater relative to those in the shopping mall effluent emphasize the disproportionate contribution of healthcare facilities to the environmental load of pharmaceutical residues. This observation aligns with previous research identifying hospitals as major point sources of antimicrobial pollution due to their concentrated and intensive pharmaceutical usage patterns [26,27]. Of note, not all ARGs detected in this study may be clinically significant. Some ARGs could be derived from intrinsic resistance of environmental organisms which rarely cause human infection [28]. Conversely, ARGs associated with clinical importance as well as horizontal transfer require specific attention (e.g., *bla*_CTX-M_ and *bla*_IMP_ groups) [29].

Compound-specific comparison of clarithromycin and levofloxacin further highlighted the above-specified differences. Levofloxacin exhibited consistently higher and more stable hospital-to-mall concentration ratios, frequently exceeding 2.0 log_10_ (equivalent to roughly 100-fold higher concentrations in hospital effluents). This pattern reflects the antibiotic’s broad-spectrum activity and primary role in the treatment of nosocomial infections, including respiratory and urinary tract diseases [30,31]. Conversely, clarithromycin exhibited lower and more variable ratios, with log_10_ values frequently below 1.0 but some falling below 0.0, indicating comparable or even higher prevalence in commercial wastewater during certain periods. This suggests that clarithromycin is widely used in both hospital and clinical settings, possibly due to its availability for outpatient use [32,33].

Temporal trends (Figure 2a) were similarly divergent for the two compounds. levofloxacin exhibited sustained elevated ratios over the entire monitoring period, whereas clarithromycin showed irregular peaks and decreased ratios. These contrasting temporal and distributional profiles underscore the differing pharmacological applications and usage behaviors of the two antimicrobials and demonstrate the capability of wastewater surveillance to capture compound-specific patterns with temporal resolution.

Distribution and density distribution analyses (Figure 2b and Figure S2) reinforced the above-mentioned interpretations. Levofloxacin showed a right-skewed unimodal distribution centered between 1.5 and 2.0 log_10_ (approximately 30–100-fold in absolute terms), consistent with its stable and sector-specific clinical use. Clarithromycin, in contrast, demonstrated a broader and more symmetrical distribution centered near 0.5 log_10_, suggesting a mean three-fold enrichment in hospital wastewater but with substantial temporal variability, highlighting its diffuse application across both hospital and community sources.

This study has several limitations. First, comparative analysis was conducted using wastewater samples from a single hospital and community site, which may limit the generalizability of our findings to broader regional and global contexts. Although the hospital harbored a diverse patient population as a global medical center, investigation of additional healthcare facilities with varying capacities and patient demographics is necessary to capture a more representative picture of the antibiotic resistomes in healthcare settings. Second, individual-level clinical data were not assessed in relation to the wastewater samples. As a result, we were unable to evaluate the potential associations between ARG abundance (e.g., RPKM values) and patient-specific factors, such as antibiotic usage patterns and infection prevalence. Importantly, this study extends the application of WBE beyond city-scale surveillance of population-wide antimicrobial use and infectious disease trends [34,35]. For the first time, our findings demonstrate the utility of WBE in resolving sector-level differences, for instance, between healthcare and commercial domains, by integrating concentration ratios, time series, and distributional analyses. The contrasting signatures of levofloxacin and clarithromycin provide a compelling case study for spatially resolved WBE, providing targeted insights into the sources, intensity, and consistency of antimicrobial usage.

## 4. Materials and Methods

### 4.1. Study Settings and Sample Collection

This was a longitudinal study to evaluate and compare the antibiotic resistomes in the wastewater samples from NCGM (hospital) and a shopping mall (community) in Tokyo, Japan. The hospital and shopping mall were located in different areas of Tokyo (approximately 40 km away). Untreated wastewater was collected from NCGM once a month between December 2019 and September 2023, resulting in a total of 46 samples. From the shopping mall, samples were collected three times between July 2020 and March 2021. To avoid a large influence by a single extreme result, we obtained multiple samples, The date of sampling was based on convenience sampling within the study period. During each sample collection, wastewater was collected using the Corning Easy-Grip round, plastic, storage bottles (50 mL in each bottle, Corning Inc., New York, NY, USA). In addition, 40 mL of wastewater was also filtered using the TPP Rapid Filtermax Vacuum Filtration system (TPP Techno Plastic Products AG, Trasadingen, Switzerland) with 49-cm^2^ polyethersulfone 0.2-µm membranes. One-quarter of the collected membranes were cut into small pieces with a sterile scalpel; this corresponds to the use of a membrane to capture bacteria from 10 mL of raw wastewater for metagenomic analysis. All small pieces were put into 0.1 and 0.5 mm ZR-96 Bashing Bead lysis tubes (Zymo Inc., Irvine, CA, USA). The tubes were frozen at −25 °C following the addition of 800 µL of the Roche bacterial lysis buffer.

The tubes were subjected to bead-beating using GenoGrinder 2010 (SPEX SamplePrep, Metuchen, NJ, USA) at 1500 rpm for 10 min. DNA purification was conducted using Roche MagnaPure (MagnaPure compact isolation: Elution step, 50 µL, Roche, Basel, Switzerland). For the preparation of DNA-seq libraries, we used the QIAseq FX DNA library kit with four cycles of enrichment (Qiagen, Hilden, Germany). For next-generation sequencing, we used the NexSeq 500 platform (150-mer; single-end; Illumina, San Diego, CA, USA). All raw read sequence files are available in the DNA Data Bank of Japan Sequence Read Archive/Sequence Read Archive database (accession numbers: DRR680724–DRR680775 [Table S5]).

### 4.2. Antimicrobial Resistome Analysis

ARG abundance was quantified using the reads per kilobase per million mapped reads (RPKM) values and ARGs-OAP v3.2.1 against the implemented ARG database to evaluate the monthly changes in the RPKM values of ARGs in the 33 hospital wastewater samples over time [13]. A generalized linear model with gamma distribution and identity links was applied to the mean RPKM values of ARGs classified by the antimicrobial drug class, with time (by month) as the independent variable. Statistical significance was set at *p* < 0.01 for regression analysis.

RPKM values of all ARGs in the hospital and shopping mall samples were compared using the two-tailed Student’s *t*-test. RPKM values for the hospital and shopping mall samples were compared using thresholds for statistical significance of *p* < 0.01 (−log_10_ *p*-value > 2) and fold change (FC) > 2 (|log_10_ FC| > 0.3). FC was calculated by dividing mean RPKM for the hospital by that for the shopping mall. We used Microsoft Excel 2019, R version 4.5.0, and Stata 18.0 for all statistical analyses.

### 4.3. Evaluation of Residual Antimicrobial Concentrations

A total of 17 antimicrobials were evaluated in the following five categories: *β*-lactams (ampicillin, benzylpenicillin, cefdinir, cefpodoxime, cefpodoxime proxetil, and ceftiofur), new quinolones (ciprofloxacin, enrofloxacin, and levofloxacin), macrolides (azithromycin and clarithromycin), tetracyclines (chlortetracycline, doxycycline, minocycline, oxytetracycline, and tetracycline), and glycopeptides (vancomycin). The compounds were selected based on their reported prevalence in hospital wastewater, wastewater treatment plants, and surface water systems in Japan and worldwide as well as their usage patterns in Japanese medical settings. All analytical standards were of high purity (>98%) [36,37,38,39,40].

Target antimicrobials in hospital wastewater samples were quantified via solid-phase extraction, followed by ultra-performance liquid chromatography–tandem mass spectrometry, as described in previous studies [41,42]. Briefly, 10 mL of each wastewater sample was filtered through the TPP Rapid Filtermax Vacuum Filtration system, as described in Section 4.1, and the filtrate was passed through the OASIS HLB solid-phase extraction cartridges (200 mg, 6 mL; Waters Corp., Milford, MA, USA) at a flow rate of 1 mL/min. The cartridges were preconditioned by washing with 6 mL of Milli-Q water adjusted to pH 3 and subsequently dried using a vacuum pump. The adsorbed analytes were eluted sequentially with 3 mL of acetone and 3 mL of methanol, followed by gentle evaporation under a nitrogen stream at 37 °C. The dried residue was reconstituted in 200 μL of a 90:10 (*v*/*v*) mixture of 0.1% formic acid in methanol. A 10 μL aliquot was injected into the ultra-performance liquid chromatography–tandem mass spectrometry system equipped with the BEH C_18_ column (2.1 mm × 100 mm, 1.7 µm; Waters Corp.) and coupled to a tandem quadrupole detector (Waters Corp.).

Quantification was performed by subtracting the blank values from those of the spiked sample solutions to correct for matrix effects and extraction losses [43,44]. Limits of detection and quantification were defined as signal-to-noise ratios of 3 and 10, respectively [45,46].

## 5. Conclusions

In conclusion, our study showed that the hospital wastewater system had more abundant ARGs, especially those associated with aminoglycosides and *β*-lactam antibiotics including carbapenems, compared to the community wastewater system. On the other hand, ARGs associated with the frequently detected AROs (e.g., ESBL-producing *Enterobacteriaceae*) in the clinical samples in Japan were detected in both the hospital and community samples, without any significant difference in the number of detected ARGs. Further investigations are necessary to outline strategies for the best use of the antibiotic resistome data from hospital and community wastewater systems. This study also introduces an application of WBE for inferring the therapeutic specificity of antimicrobials by comparing the hospital and community effluents. Integration of log-transformed ratios with temporal and statistical characterization provides a valuable framework for tracking pharmaceutical usage behaviors and supporting environmental monitoring, antimicrobial stewardship, and regulatory policy development.

## Figures and Tables

**Table 1:** List of antimicrobial resistant genes with significantly higher RPKM (−log_10_ *p*-value > 2.0 and log_10_ FC > 0.3) in the hospital samples compared with the shopping mall samples.

Antimicrobial Class	Antimicrobial Resistant Gene	−log_10_(*p*)	log_10_(FC)
*β*-lactam	*bla*_IMP-1_	7.8	1.4
*β*-lactam	*bla*_OXA-10_	7.5	1.4
*β*-lactam	*bla*_NPS-1_	6.7	1.3
*β*-lactam	*bla*_GES-15_	6.3	1.8
Aminoglycoside	*aph(3″)-Ib*	6.3	0.5
Aminoglycoside	*aac(6′)-Ib′*	6.2	1.3
Aminoglycoside	*aac(6′)-Ib-cr*	6.0	1.2
*β*-lactam	*bla*_IMP-55_	4.9	1.3
Multi-drug	*oprC*	4.7	1.1
Aminoglycoside	*aac(6′)-Ie-aph(2″* *)-Ia*	4.7	1.2
Aminoglycoside	*aac(6′)-Ib11*	4.3	1.0
*β*-lactam	*bla*_IMP-42_	4.2	1.0
Aminoglycoside	*aac(6′)-Ib8*	3.8	1.1
*β*-lactam	*bla*_CfxA6_	3.3	1.0
Macrolide-lincosamide-streptogramin	*ere(A)*	3.3	0.9
Aminoglycoside	*aadA17*	3.1	0.9
Multi-drug	*bpeF*	2.8	0.9
Multi-drug	*qacEΔ1*	2.6	0.6
Aminoglycoside	*aadS*	2.4	0.6
Multi-drug	*mdsB*	2.4	1.1
Aminoglycoside	*aph(6)-Id*	2.4	0.4
Tetracycline	*tet*(M)	2.1	0.6
*β*-lactam	*bla*_CfxA_	2.1	0.9

Abbreviations: FC; fold change, RPKM; reads per kilobase per million.

## Data Availability

All raw read sequence files are available in the DNA Data Bank of Japan Sequence Read Archive (DRA)/Sequence Read Archive (SRA) database (accession numbers: DRR680724–DRR680775 [Table S5] available of URLs from https://www.ncbi.nlm.nih.gov/search/all/?term=DRR680724 to https://www.ncbi.nlm.nih.gov/search/all/?term=DRR680775), accessed on 14 January 2026. The dataset on ARG abundance in hospital and community wastewater samples is available and detailed in Table S1 of the Supplementary Information. Residual antimicrobial concentrations in hospital wastewater are documented in Table S4 of the Supplementary Information.

## Supplementary Materials

The following supporting information can be downloaded at: https://www.mdpi.com/article/10.3390/antibiotics15010099/s1, Supplemental Figure S1: Mean RPKM by antimicrobial class in monthly hospital wastewater samples between December 2019 and September 2023; Supplemental Figure S2. Probability density functions of the log_10_ concentration ratios of clarithromycin and levofloxacin from June 2020 to June 2023; Supplemental Table S1: Mean RPKM of wastewater samples in the hospital and shopping mall; Supplemental Table S2: Sampling date of wastewater samples; Supplemental Table S3: Generalized liner model to evaluate association between mean RPKM by antimicrobial class and monthly trend; Supplemental Table S4: Temporal variations in the ratio of clarithromycin and levofloxacin in wastewater from a hospital compared to a shopping mall; Supplemental Table S5: All raw read sequence files in the DRA/SRA database.

## Abbreviations

The following abbreviations are used in this manuscript:

AMR	Antimicrobial resistance
ARG	Antimicrobial resistance gene
TLA	Carbapenemase-producing *Enterobacteriaceae*
CRE	Carbapenem-resistant *Enterobacteriaceae*
ESBL	Extended spectrum *β*-lactamase
FC	Fold change
NCGM	National Center for Global Health and Medicine
RPKM	reads per kilobase per million mapped reads
WBE	wastewater-based epidemiology
