# Supplementary material for: Environmental Dissemination of Antimicrobial Resistance: A Resistome-Based Comparison of Hospital and Community Wastewater Sources
Source: Antibiotics (Basel). 2026 Jan 19;15(1):99. doi: 10.3390/antibiotics15010099 (PMC12838039; doi:10.3390/antibiotics15010099)

Supplemental Figure S1. Mean RPKM by antimicrobial class in monthly hospital wastewater samples between December 2019 and September 2023.

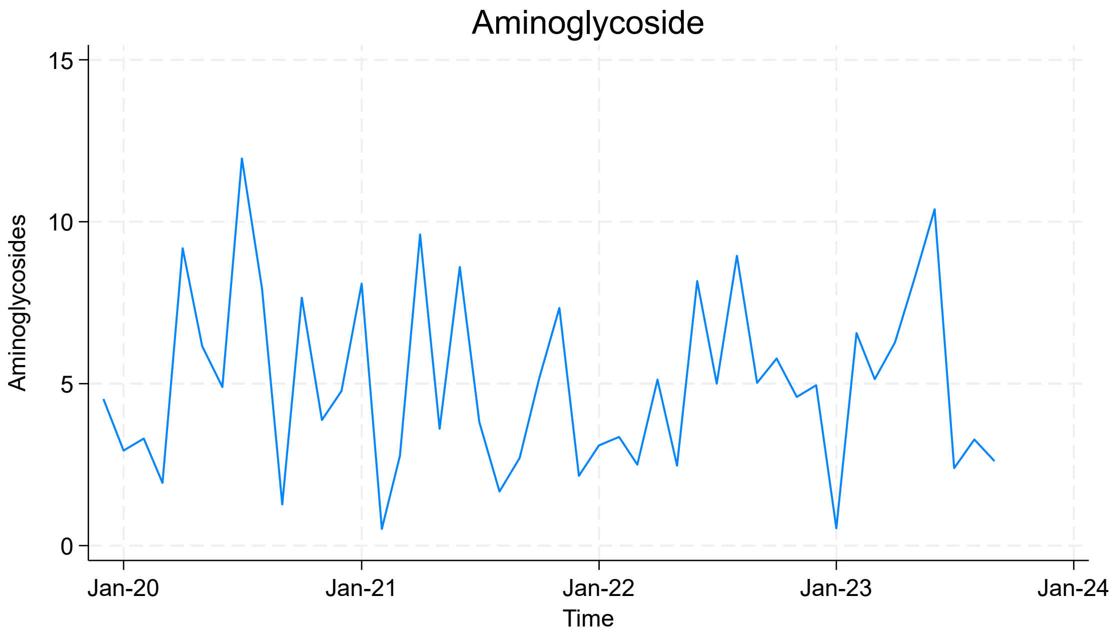

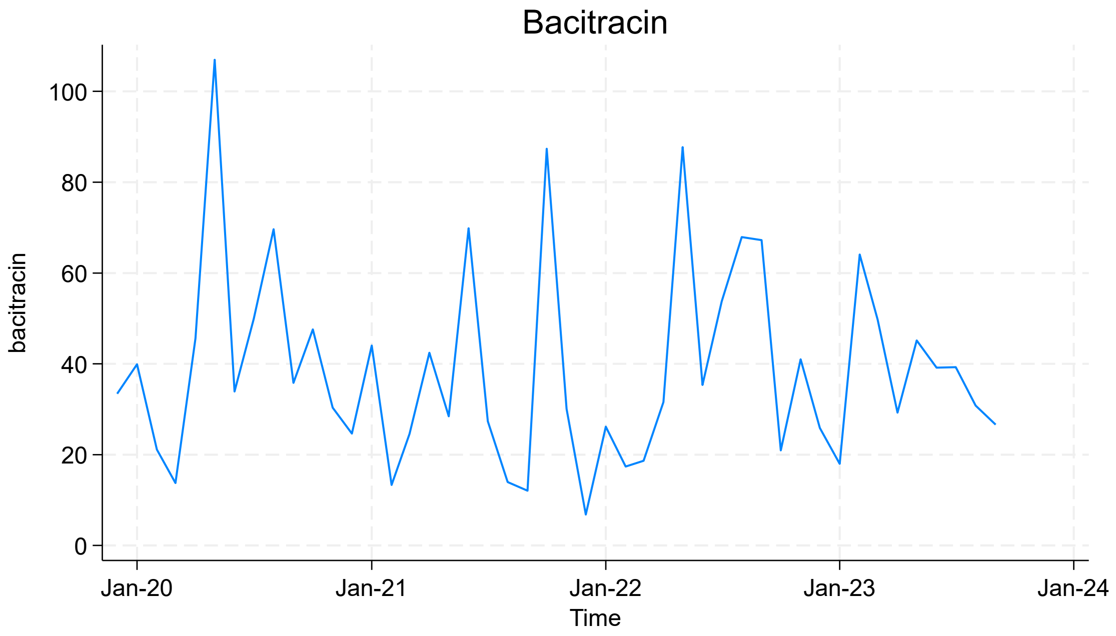

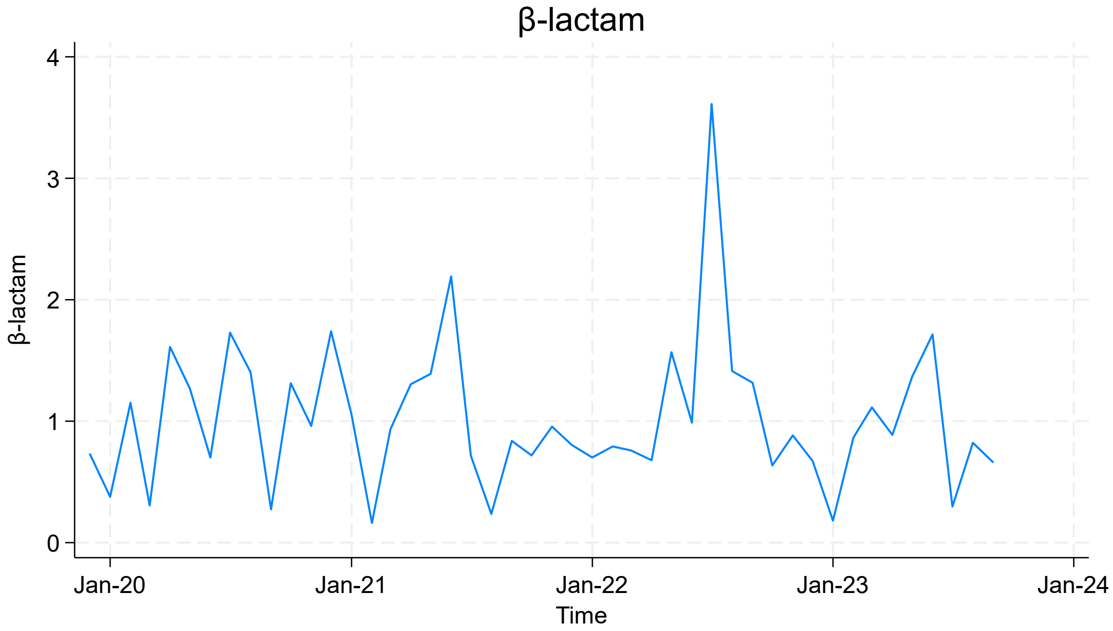
β-lactam
β-lactam
0
1
2
3
4
Jan-20
Jan-21
Jan-22
Jan-23
Jan-24
Time

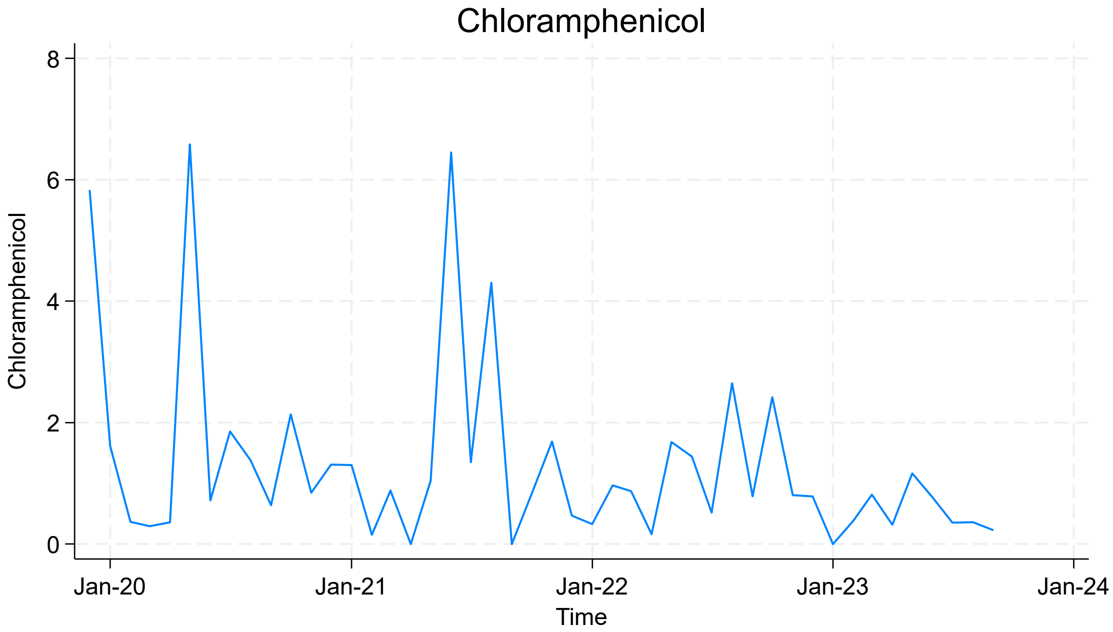
Chloramphenicol
Chloramphenicol
0
2
4
6
8
Jan-20
Jan-21
Jan-22
Jan-23
Jan-24
Time

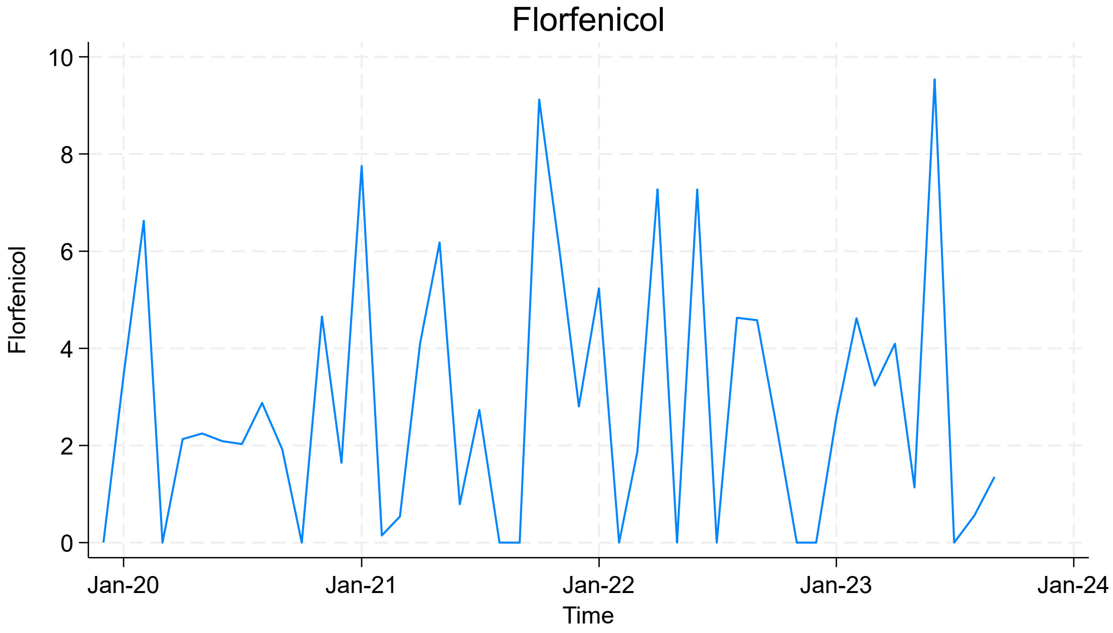

Florfenicol
10
8
6
4
2
0
Florfenicol
Jan-20
Jan-21
Jan-22
Jan-23
Jan-24
Time

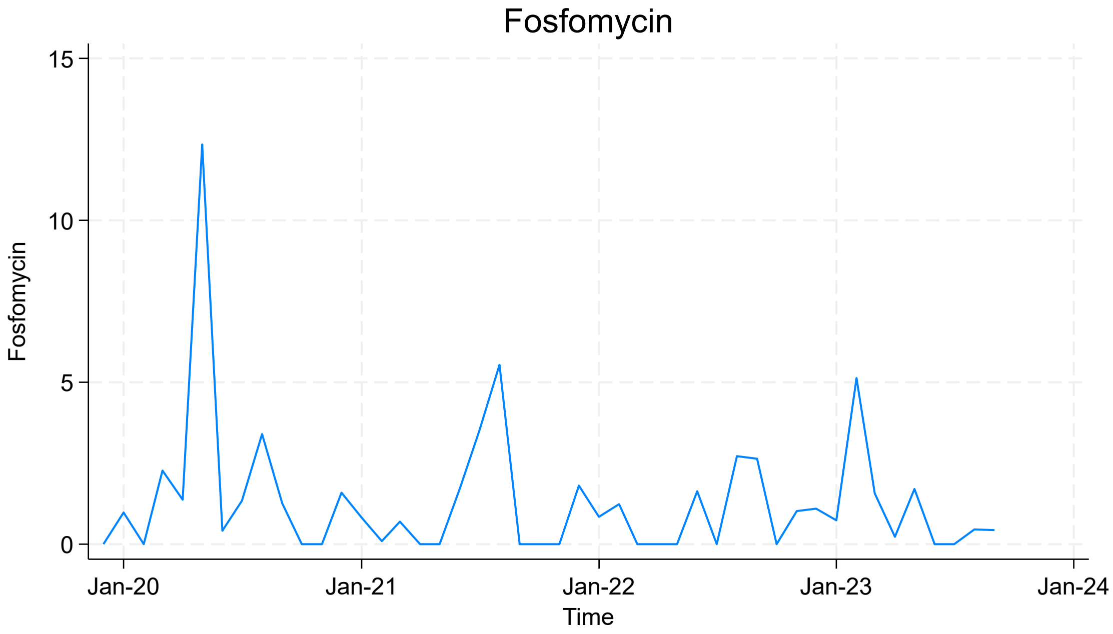

Fosfomycin
15
10
5
0
Fosfomycin
Jan-20
Jan-21
Jan-22
Jan-23
Jan-24
Time

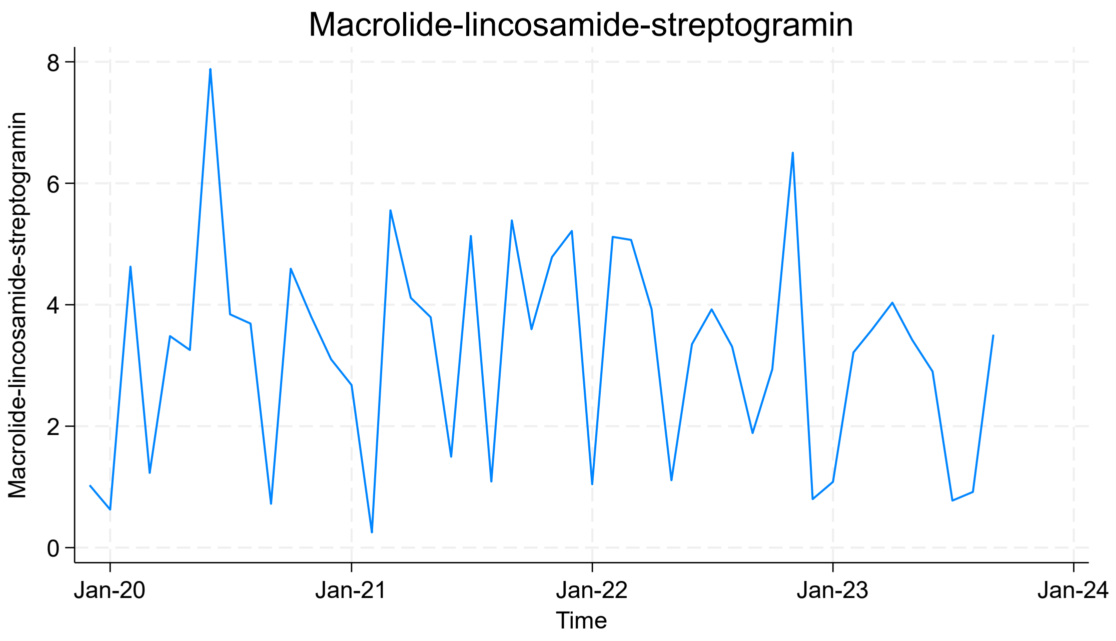
Macrolide-lincosamide-streptogramin
Macrolide-lincosamide-streptogramin
0
2
4
6
8
Jan-20
Jan-21
Jan-22
Jan-23
Jan-24
Time

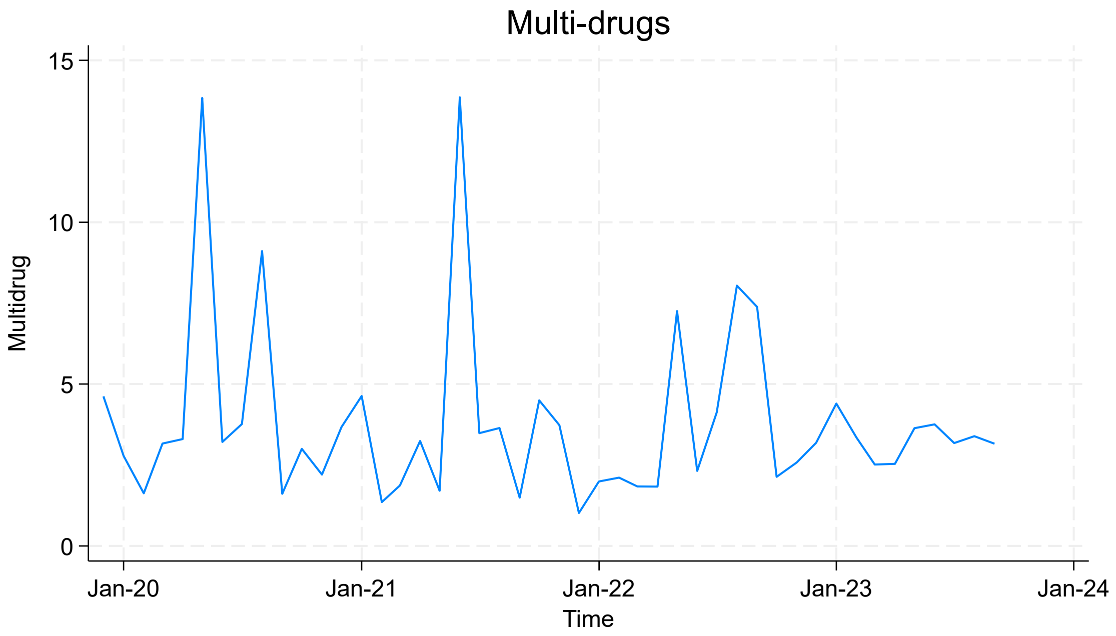
Multi-drugs
Multidrug
0
5
10
15
Jan-20
Jan-21
Jan-22
Jan-23
Jan-24
Time

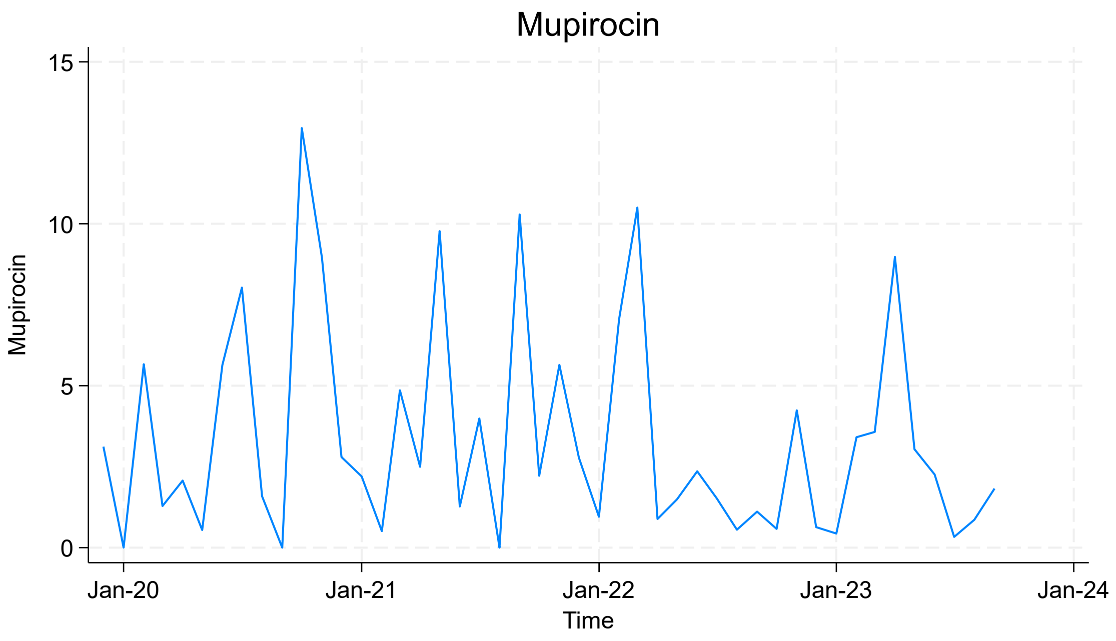
Mupirocin
Mupirocin
15
10
5
0
Jan-20
Jan-21
Jan-22
Jan-23
Jan-24
Time

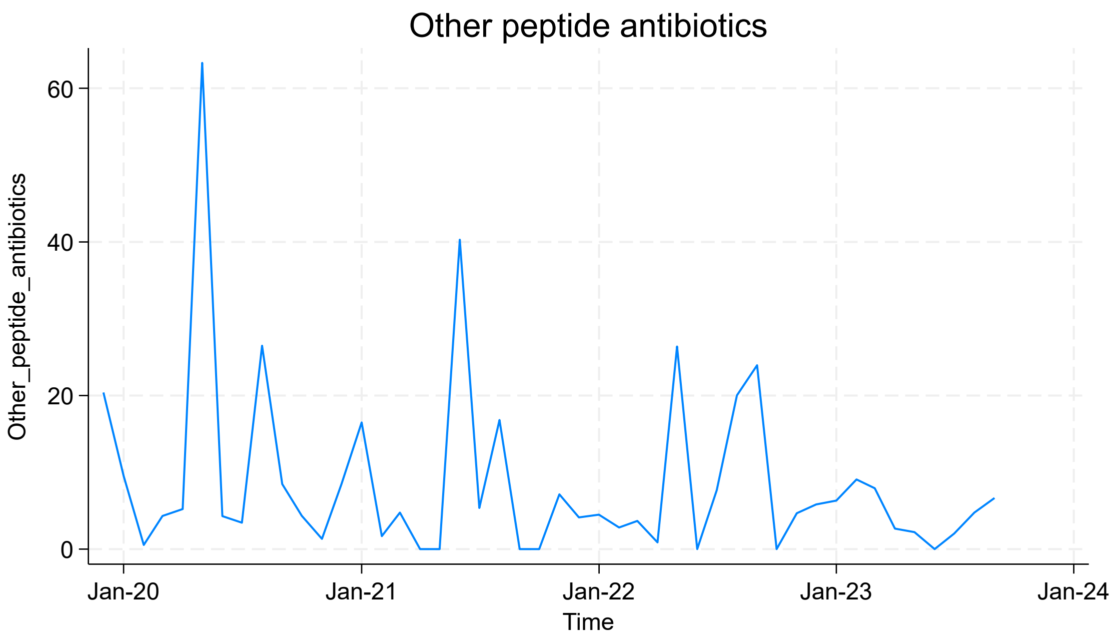
Other peptide antibiotics
Other_peptide_antibiotics
60
40
20
0
Jan-20
Jan-21
Jan-22
Jan-23
Jan-24
Time

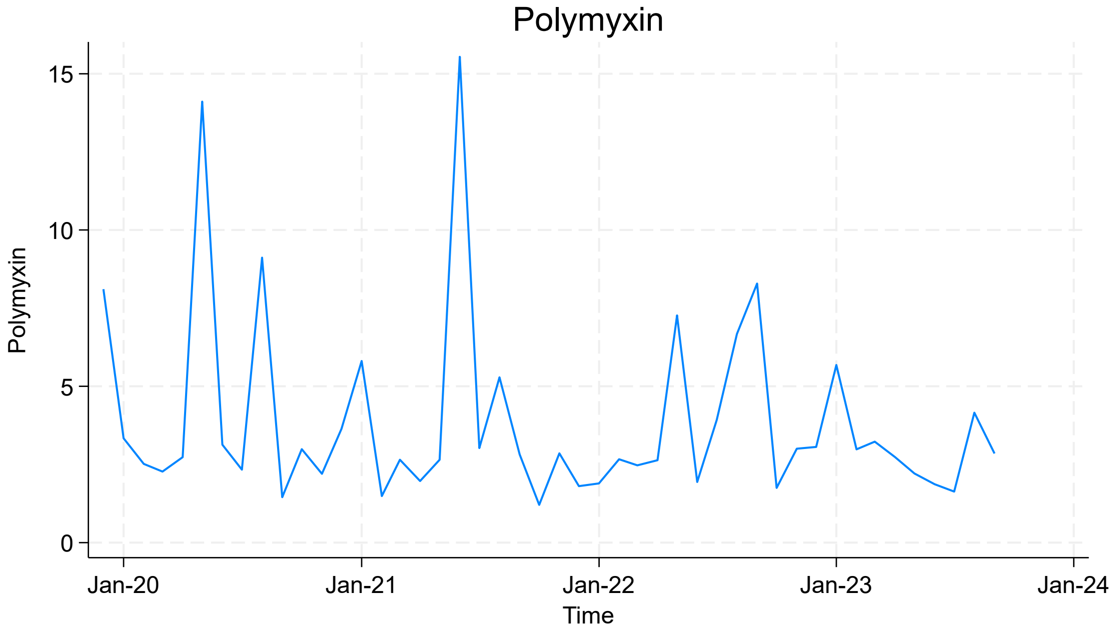
Polymyxin
15
10
5
0
Polymyxin
Jan-20
Jan-21
Jan-22
Jan-23
Jan-24
Time

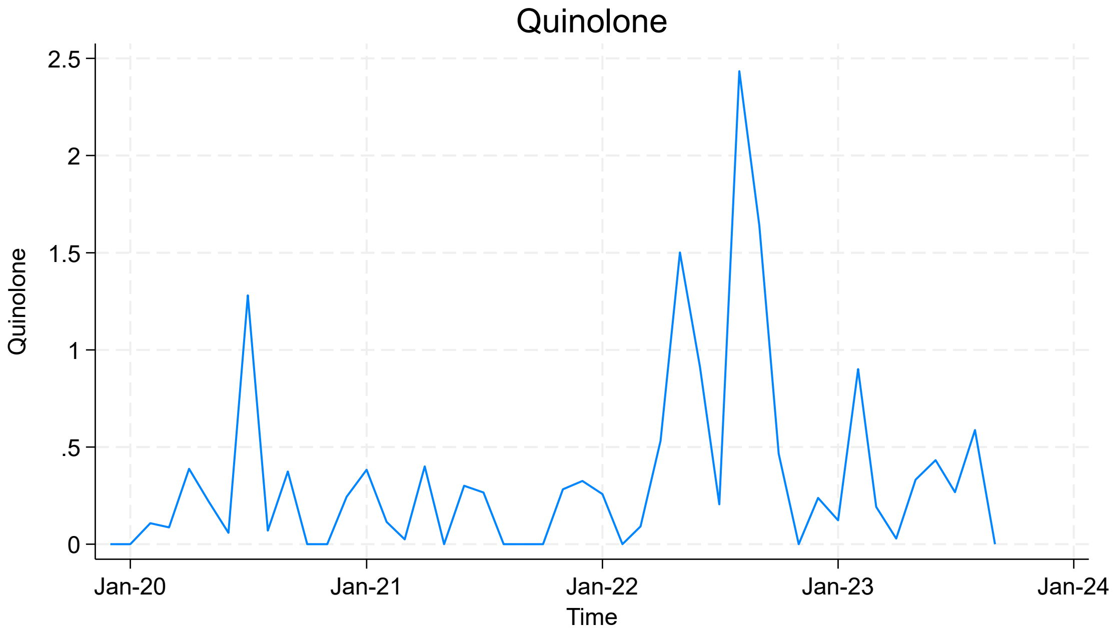
Quinolone
2.5
2
1.5
1
.5
0
Quinolone
Jan-20
Jan-21
Jan-22
Jan-23
Jan-24
Time

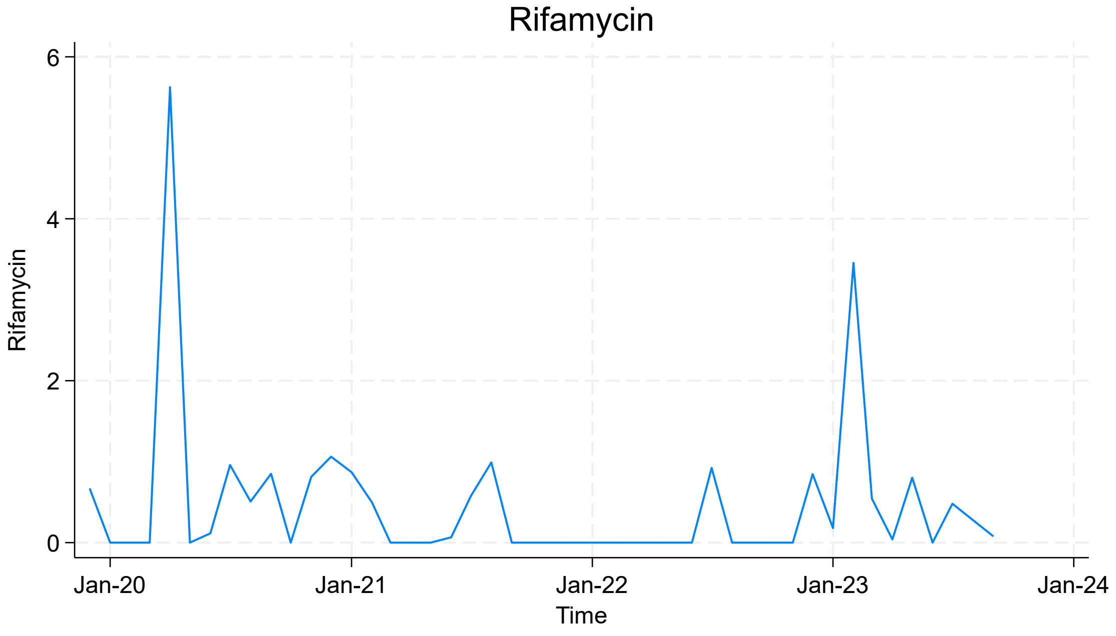
Rifamycin
Rifamycin
6
4
2
0
Jan-20
Jan-21
Jan-22
Jan-23
Jan-24
Time

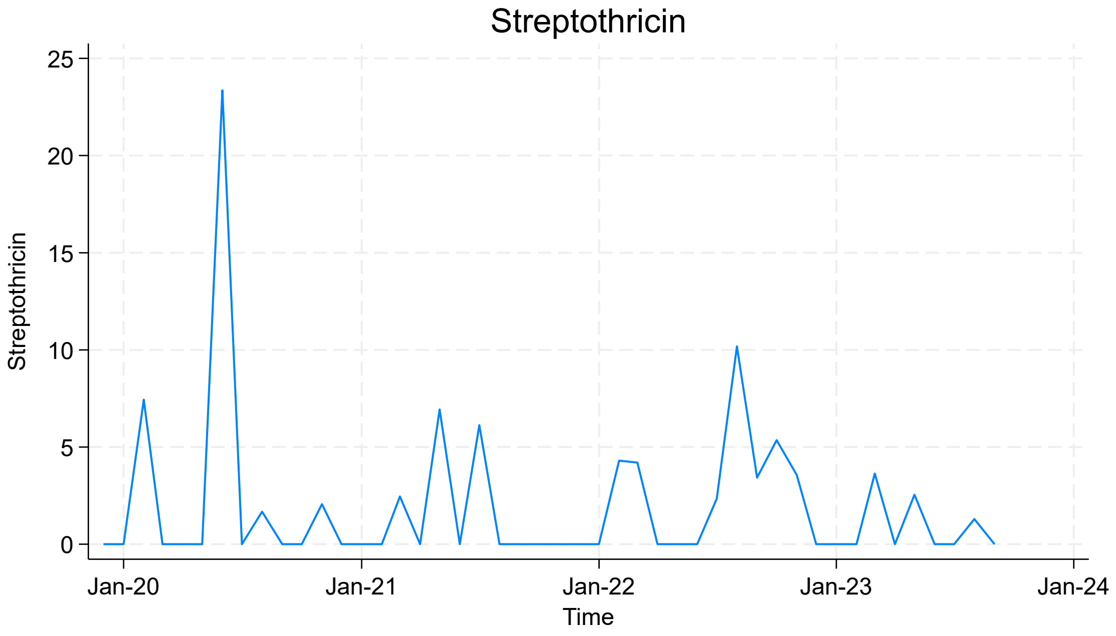
Streptothricin
Streptothricin
25
20
15
10
5
0
Jan-20
Jan-21
Jan-22
Jan-23
Jan-24
Time

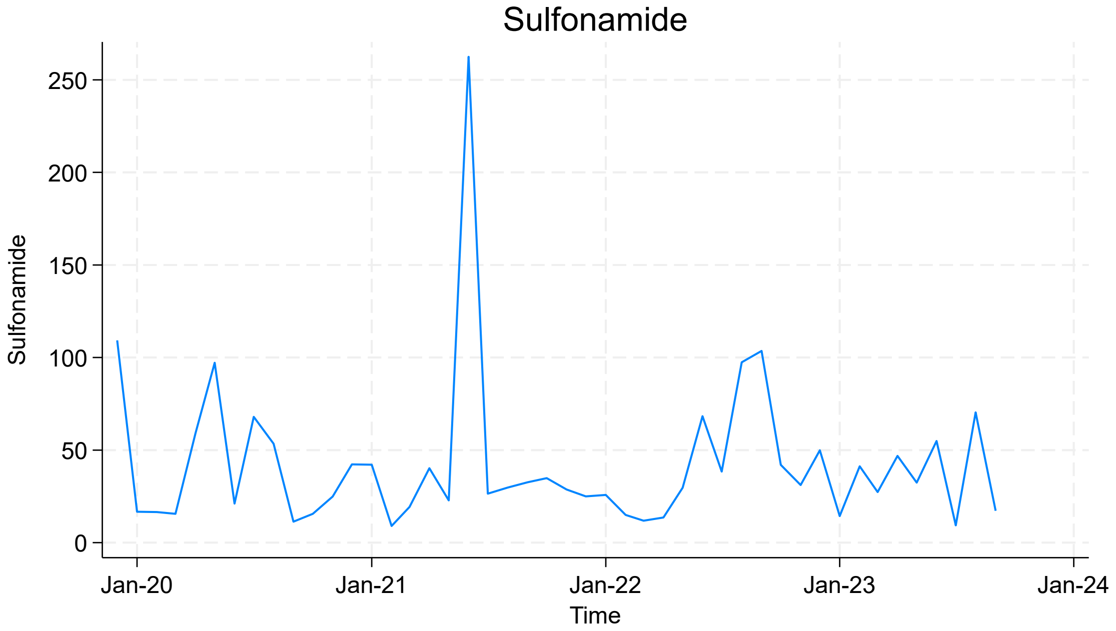
Sulfonamide
Sulfonamide
0
50
100
150
200
250
Jan-20
Jan-21
Jan-22
Jan-23
Jan-24
Time

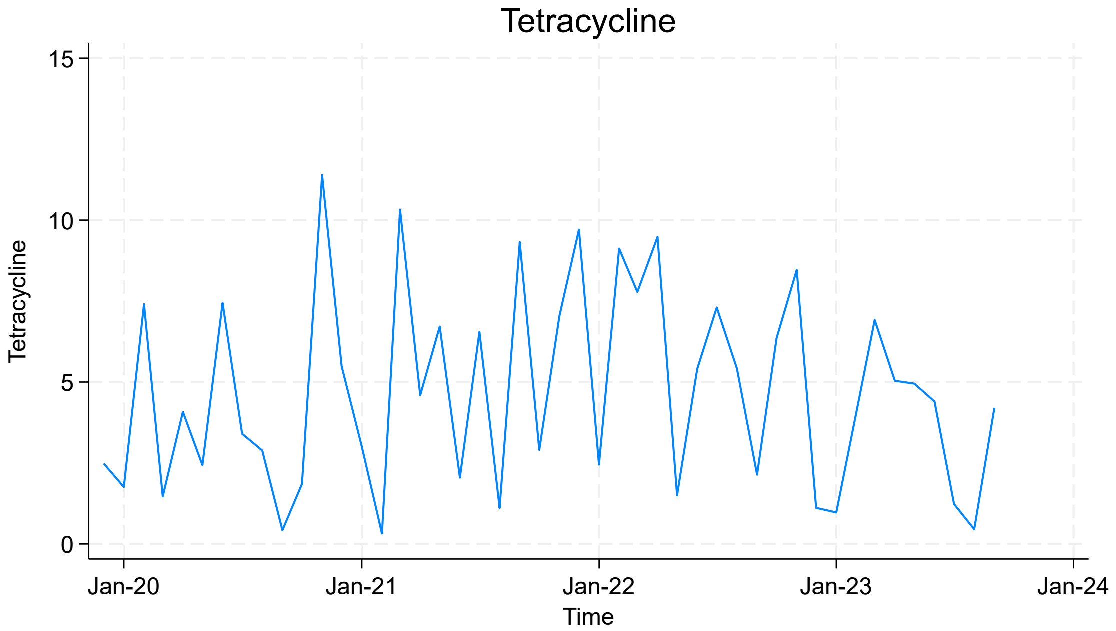
Tetracycline
Tetracycline
0
5
10
15
Jan-20
Jan-21
Jan-22
Jan-23
Jan-24
Time

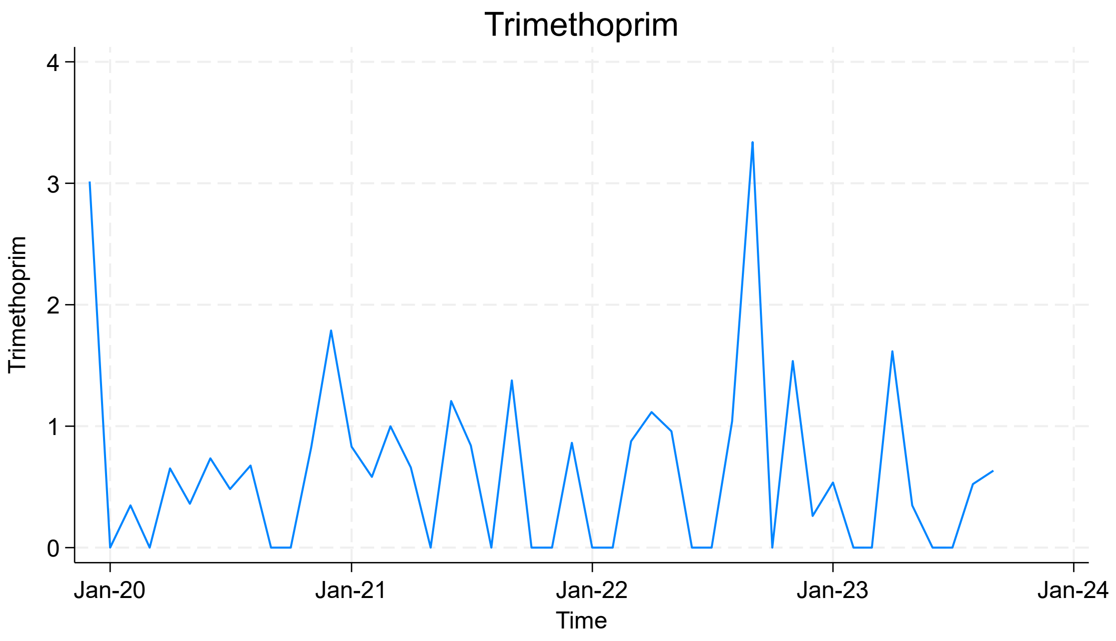

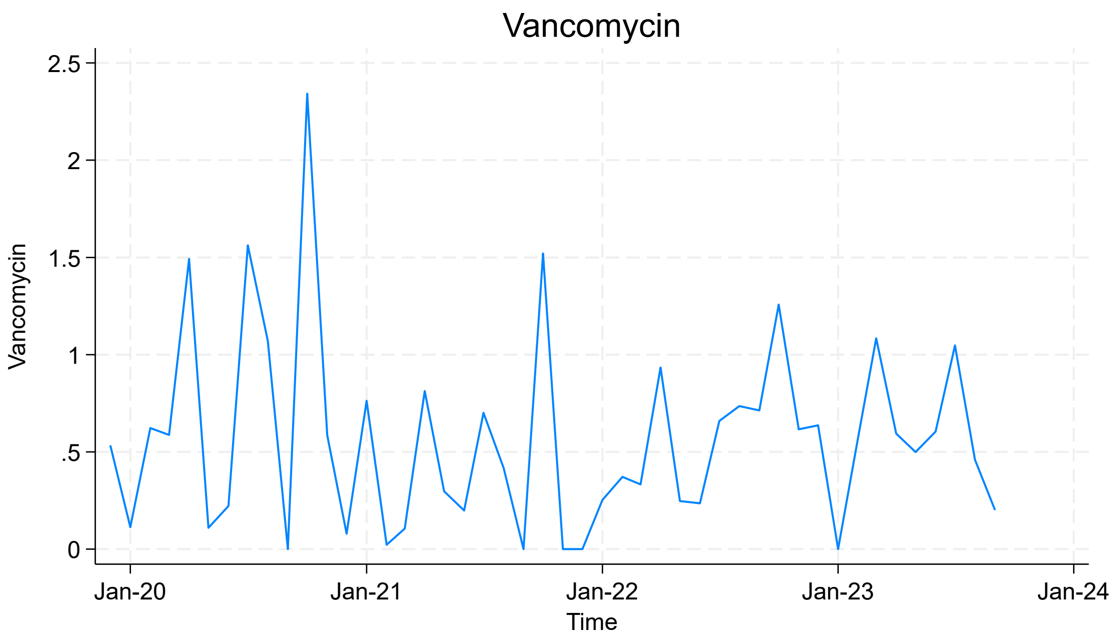

The mean RPKM by drug class was calculated by the average of RPKM of ARGs detected either by hospital or community samples.

Supplemental Figure S2. Probability density functions of the $\log_{10}$ concentration ratios of clarithromycin and levofloxacin from June 2020 to June 2023.

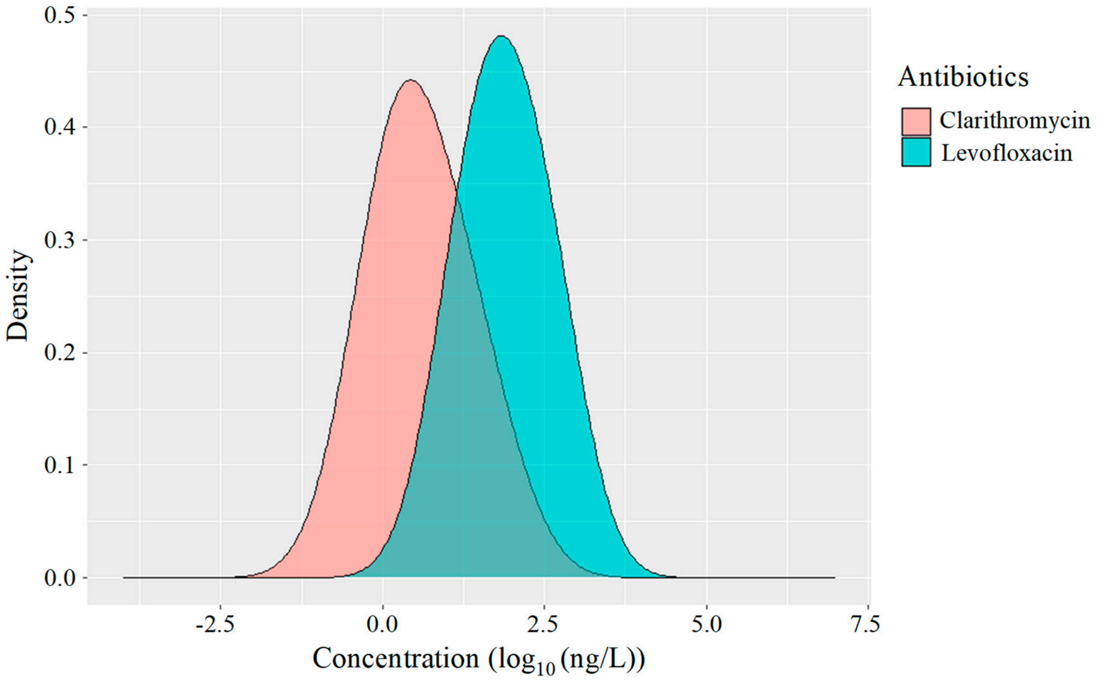

Supplement: Supplementary file 1 [file antibiotics-15-00099-s001.zip › Supplemental Figures.pdf]
