# Supplementary material for: Environmental Dissemination of Antimicrobial Resistance: A Resistome-Based Comparison of Hospital and Community Wastewater Sources
Source: Antibiotics (Basel). 2026 Jan 19;15(1):99. doi: 10.3390/antibiotics15010099 (PMC12838039; doi:10.3390/antibiotics15010099)
Supplement: Supplementary file 1 [file antibiotics-15-00099-s001.zip › Supplemental Tables_Antibiotics.pdf]

Supplemental Table S1. Mean RPKM of wastewater samples in the hospital and shopping mall

**ARGs subtype**	Hospital mean RPKM Mean	Shopping mall mean RPKM Mean	-log10 (p)	log10(FC)
**aminoglycoside__AAC(2')-Ib**	0.6	0.0	1.6	
**aminoglycoside__AAC(2')-Ic**	0.3	0.0	0.6	
**aminoglycoside__AAC(3)-Ic**	0.0	0.0	0.5	
**aminoglycoside__AAC(3)-IIc**	0.3	0.0	0.8	
**aminoglycoside__AAC(3)-IId**	0.7	1.6	0.2	-0.3
**aminoglycoside__AAC(3)-IIe**	0.4	0.9	0.2	-0.3
**aminoglycoside__AAC(3)-IIIb**	0.2	0.0	0.8	
**aminoglycoside__AAC(3)-IV**	0.1	0.0	0.5	
**aminoglycoside__AAC(3)-IVb**	0.0	0.0	0.5	
**aminoglycoside__AAC(6')-29b**	0.4	0.0	0.5	
**aminoglycoside__AAC(6')-30/AAC(6')-Ib' fusion protein**	0.2	0.0	0.9	
**aminoglycoside__AAC(6')-31**	19.3	0.0	3.0	
**aminoglycoside__AAC(6')-32**	0.7	0.0	1.7	
**aminoglycoside__AAC(6')-Ia**	4.8	0.0	2.2	
**aminoglycoside__AAC(6')-Iae**	0.1	0.0	0.5	
**aminoglycoside__AAC(6')-Iaf**	0.1	0.0	0.6	
**aminoglycoside__AAC(6')-Ib**	5.4	1.0	1.8	0.7
**aminoglycoside__AAC(6')-Ib'**	19.1	1.0	6.2	1.3
**aminoglycoside__AAC(6')-Ib10**	7.1	5.7	0.1	0.1
**aminoglycoside__AAC(6')-Ib11**	20.9	1.9	4.3	1.0
**aminoglycoside__AAC(6')-Ib3**	0.5	0.0	1.2	
**aminoglycoside__AAC(6')-Ib4**	3.3	0.0	4.7	
**aminoglycoside__AAC(6')-Ib7**	3.9	0.0	5.3	
**aminoglycoside__AAC(6')-Ib8**	5.1	0.4	3.8	1.1
**aminoglycoside__AAC(6')-Ib9**	14.7	4.6	1.0	0.5
**aminoglycoside__AAC(6')-Ib-cr**	33.3	2.3	6.0	1.2

ARGs subtype	Hospital mean RPKM Mean	Shopping mall mean RPKM Mean	-log10 (p)	log10(FC)
**aminoglycoside__AAC(6')-Ib-Hangzhou**	5.8	1.1	1.9	0.7
**aminoglycoside__AAC(6')-Ib-Suzhou**	3.3	0.5	1.8	0.8
**aminoglycoside__AAC(6')-Ie-APH(2'')-Ia**	3.2	0.2	4.7	1.2
**aminoglycoside__AAC(6')-Ig**	0.0	0.0	0.5	
**aminoglycoside__AAC(6')-Ii**	0.2	0.0	0.5	
**aminoglycoside__AAC(6')-IIa**	5.0	0.0	3.4	
**aminoglycoside__AAC(6')-IIb**	0.1	0.0	0.6	
**aminoglycoside__AAC(6')-IIc**	2.1	0.0	3.2	
**aminoglycoside__AAC(6')-Il**	13.9	0.0	2.0	
**aminoglycoside__AAC(6')-Im**	0.2	0.0	1.0	
**aminoglycoside__AAC(6')-Is**	0.1	0.0	0.5	
**aminoglycoside__aad(6)**	5.7	12.5	0.2	-0.3
**aminoglycoside__aadA**	27.8	11.2	1.0	0.4
**aminoglycoside__aadA10**	0.9	0.0	2.6	
**aminoglycoside__aadA11**	1.7	0.0	2.3	
**aminoglycoside__aadA12**	5.6	1.7	0.9	0.5
**aminoglycoside__aadA13**	3.0	0.0	3.8	
**aminoglycoside__aadA15**	0.1	0.0	0.8	
**aminoglycoside__aadA16**	0.6	0.3	0.2	0.3
**aminoglycoside__aadA17**	3.0	0.3	3.1	0.9
**aminoglycoside__aadA2**	3.0	1.0	0.7	0.5
**aminoglycoside__aadA21**	1.6	3.7	0.3	-0.4
**aminoglycoside__aadA22**	4.1	3.0	0.1	0.1
**aminoglycoside__aadA23**	10.7	6.8	0.7	0.2
**aminoglycoside__aadA24**	1.5	0.0	3.6	
**aminoglycoside__aadA25**	0.7	0.0	1.9	
**aminoglycoside__aadA27**	1.2	4.0	0.5	-0.5
**aminoglycoside__aadA3**	5.5	4.4	0.2	0.1
**aminoglycoside__aadA4**	0.5	0.0	0.8	
**aminoglycoside__aadA5**	47.3	14.1	1.4	0.5

**ARGs subtype**	Hospital mean RPKM Mean	Shopping mall mean RPKM Mean	-log10 (p)	log10(FC)
**aminoglycoside__aadA6**	1.4	0.0	1.5	
**aminoglycoside__aadA6/aadA10**	5.5	2.8	0.4	0.3
**aminoglycoside__aadA7**	1.4	0.0	3.1	
**aminoglycoside__aadA8**	3.0	1.3	0.7	0.3
**aminoglycoside__aadA8b**	1.8	0.3	1.5	0.7
**aminoglycoside__aadK**	0.1	0.3	0.2	-0.5
**aminoglycoside__aadS**	22.8	5.6	2.4	0.6
**aminoglycoside__amrB**	0.0	0.1	0.0	-0.1
**aminoglycoside__ANT(2")-Ia**	3.4	2.8	0.1	0.1
**aminoglycoside__ANT(3")-IIa**	0.3	0.0	0.9	
**aminoglycoside__ANT(3")-Ii-AAC(6')-IId fusion protein**	0.1	0.0	1.0	
**aminoglycoside__ANT(3")-IIc**	0.3	0.3	0.1	-0.1
**aminoglycoside__ANT(4')-Ib**	0.1	0.0	0.5	
**aminoglycoside__ANT(6)-Ia**	1.9	0.0	2.2	
**aminoglycoside__ANT(6)-Ib**	0.9	3.4	0.4	-0.6
**aminoglycoside__APH(2")-If**	0.5	0.0	1.5	
**aminoglycoside__APH(2")-Ig**	0.1	0.0	0.6	
**aminoglycoside__APH(2")-IIa**	0.2	0.0	1.3	
**aminoglycoside__APH(3')-Ia**	3.6	2.7	0.2	0.1
**aminoglycoside__APH(3')-Ib**	0.1	1.0	0.3	-1.1
**aminoglycoside__APH(3")-Ib**	43.3	12.6	6.3	0.5
**aminoglycoside__APH(3')-IIa**	0.1	0.0	0.8	
**aminoglycoside__APH(3')-IIc**	0.1	0.0	0.5	
**aminoglycoside__APH(3')-IIIa**	5.1	0.0	2.8	
**aminoglycoside__APH(3')-VI**	0.0	0.3	0.4	
**aminoglycoside__APH(3')-VIIa**	0.0	0.0	0.5	
**aminoglycoside__APH(6)-Ic**	0.3	0.0	1.1	
**aminoglycoside__APH(6)-Id**	33.2	14.1	2.4	0.4
**aminoglycoside__aphA15**	1.3	0.0	3.1	

ARGs subtype	Hospital mean RPKM Mean	Shopping mall mean RPKM Mean	-log10 (p)	log10(FC)
**aminoglycoside__npmA**	0.8	0.0	0.6	
**antibacterial_fatty_acid__farB**	0.1	4.6	0.8	-1.9
**bacitracin__bacA**	115.6	101.5	0.2	0.1
**bacitracin__BahA**	0.0	0.0	0.6	
**bacitracin__bcrA**	1.1	0.3	0.9	0.6
**beta_lactam__ACC-1**	0.1	0.0	0.5	
**beta_lactam__ACC-4**	0.0	0.0	0.5	
**beta_lactam__ACI-1**	1.0	0.6	0.2	0.2
**beta_lactam__ACT-12**	0.0	0.0	0.5	
**beta_lactam__ACT-15**	0.0	0.0	0.5	
**beta_lactam__ACT-17**	0.0	0.0	0.5	
**beta_lactam__ACT-2**	0.0	0.0	0.5	
**beta_lactam__ACT-20**	0.0	0.0	0.5	
**beta_lactam__ACT-22**	0.2	0.0	0.6	
**beta_lactam__ACT-28**	0.1	0.0	0.9	
**beta_lactam__ACT-29**	0.3	1.8	0.3	-0.8
**beta_lactam__ACT-30**	0.1	0.0	0.5	
**beta_lactam__ACT-37**	0.1	0.0	0.5	
**beta_lactam__ACT-5**	0.1	0.0	0.5	
**beta_lactam__ACT-6**	0.0	0.2	0.4	
**beta_lactam__ACT-9**	0.1	0.0	0.5	
**beta_lactam__ADC-8**	0.4	2.4	0.5	-0.8
**beta_lactam__AER-1**	0.4	0.0	1.4	
**beta_lactam__AIM-1**	0.1	0.0	0.5	
**beta_lactam__AQU-1**	0.0	0.2	0.3	-1.0
**beta_lactam__BcI**	0.2	0.0	0.7	
**beta_lactam__BKC-1**	0.0	0.0	0.5	
**beta_lactam__blaF**	0.3	0.0	1.4	
**beta_lactam__CARB-1**	0.2	0.0	0.7	
**beta_lactam__CARB-14**	0.0	0.0	0.5	

**ARGs subtype**	Hospital mean RPKM Mean	Shopping mall mean RPKM Mean	-log10 (p)	log10(FC)
**beta_lactam__CARB-2**	0.4	0.0	1.4	
**beta_lactam__CARB-3**	0.0	0.0	0.5	
**beta_lactam__CARB-6**	0.4	0.0	1.3	
**beta_lactam__CAU-1**	0.2	0.0	0.8	
**beta_lactam__CblA-1**	10.7	11.7	0.1	0.0
**beta_lactam__CcrA beta-lactamase**	0.2	0.0	0.9	
**beta_lactam__cepA**	8.8	1.8	1.6	0.7
**beta_lactam__CepS**	0.1	1.4	0.4	-1.2
**beta_lactam__CFE-1**	0.0	0.0	0.6	
**beta_lactam__CfxA**	2.5	0.3	2.1	0.9
**beta_lactam__CfxA2**	42.7	13.8	0.8	0.5
**beta_lactam__CfxA3**	9.8	9.3	0.0	0.0
**beta_lactam__CfxA4**	3.3	1.4	0.5	0.4
**beta_lactam__CfxA5**	11.2	4.8	0.5	0.4
**beta_lactam__CfxA6**	11.2	1.1	3.3	1.0
**beta_lactam__CGA-1**	0.1	0.0	0.5	
**beta_lactam__Chryseobacterium meningosepticum BlaB**	0.1	0.0	0.5	
**beta_lactam__CMH-1**	0.1	3.7	0.4	-1.6
**beta_lactam__CMY-1**	0.0	0.0	0.6	
**beta_lactam__CMY-10**	0.0	0.0	0.5	
**beta_lactam__CMY-100**	0.1	0.2	0.2	-0.3
**beta_lactam__CMY-101**	0.2	0.0	1.0	
**beta_lactam__CMY-103**	0.0	0.0	0.5	
**beta_lactam__CMY-104**	0.0	0.0	0.5	
**beta_lactam__CMY-108**	0.0	0.0	0.5	
**beta_lactam__CMY-11**	0.1	0.0	0.7	
**beta_lactam__CMY-110**	0.0	0.0	0.5	
**beta_lactam__CMY-112**	0.1	0.2	0.1	-0.2
**beta_lactam__CMY-113**	0.1	0.0	1.0	

**ARGs subtype**	Hospital mean RPKM Mean	Shopping mall mean RPKM Mean	-log10 (p)	log10(FC)
**beta_lactam__CMY-115**	0.0	0.2	0.3	-1.1
**beta_lactam__CMY-117**	0.0	0.0	0.5	
**beta_lactam__CMY-119**	0.0	0.0	0.8	
**beta_lactam__CMY-135**	0.2	0.0	1.0	
**beta_lactam__CMY-19**	1.0	0.0	2.3	
**beta_lactam__CMY-26**	0.1	0.0	1.0	
**beta_lactam__CMY-34**	0.0	0.0	0.7	
**beta_lactam__CMY-37**	0.0	0.0	0.5	
**beta_lactam__CMY-39**	0.0	0.0	0.7	
**beta_lactam__CMY-4**	0.0	0.0	0.5	
**beta_lactam__CMY-41**	0.3	0.0	1.0	
**beta_lactam__CMY-42**	0.0	0.0	0.5	
**beta_lactam__CMY-45**	0.0	0.0	0.5	
**beta_lactam__CMY-46**	0.0	0.0	0.5	
**beta_lactam__CMY-47**	0.0	0.0	0.8	
**beta_lactam__CMY-48**	0.6	0.5	0.1	0.1
**beta_lactam__CMY-50**	0.0	0.0	0.5	
**beta_lactam__CMY-58**	0.1	0.0	0.8	
**beta_lactam__CMY-59**	0.4	0.0	1.6	
**beta_lactam__CMY-6**	0.0	0.0	0.5	
**beta_lactam__CMY-61**	0.1	0.0	0.5	
**beta_lactam__CMY-63**	0.0	0.0	0.5	
**beta_lactam__CMY-64**	0.1	0.0	0.8	
**beta_lactam__CMY-65**	0.0	0.0	0.5	
**beta_lactam__CMY-66**	0.0	0.0	0.5	
**beta_lactam__CMY-68**	0.1	0.0	0.5	
**beta_lactam__CMY-70**	0.0	0.0	0.5	
**beta_lactam__CMY-71**	0.0	0.0	0.5	
**beta_lactam__CMY-72**	0.3	0.0	1.2	
**beta_lactam__CMY-73**	0.0	0.0	0.5	

ARGs subtype	Hospital mean RPKM Mean	Shopping mall mean RPKM Mean	-log10 (p)	log10(FC)
**beta_lactam__CMY-75**	0.0	0.0	0.5	
**beta_lactam__CMY-77**	0.1	0.0	0.9	
**beta_lactam__CMY-79**	0.2	0.0	1.3	
**beta_lactam__CMY-8**	6.7	0.0	3.1	
**beta_lactam__CMY-82**	0.1	0.0	0.7	
**beta_lactam__CMY-84**	0.2	1.8	0.3	-1.0
**beta_lactam__CMY-85**	0.1	0.0	0.8	
**beta_lactam__CMY-86**	0.0	0.0	0.5	
**beta_lactam__CMY-9**	0.9	0.0	1.5	
**beta_lactam__CMY-90**	0.1	0.0	0.7	
**beta_lactam__CMY-98**	0.1	0.0	0.8	
**beta_lactam__CMY-99**	0.0	0.0	0.8	
**beta_lactam__cphA2**	0.4	0.0	0.5	
**beta_lactam__cphA3**	0.0	0.4	0.4	
**beta_lactam__cphA6**	0.0	1.8	0.4	-1.7
**beta_lactam__cphA7**	0.3	3.5	0.5	-1.1
**beta_lactam__crxA**	0.1	0.0	0.6	
**beta_lactam__CTX-M-1**	0.2	0.0	1.1	
**beta_lactam__CTX-M-106**	0.0	0.0	0.5	
**beta_lactam__CTX-M-107**	0.1	0.0	0.5	
**beta_lactam__CTX-M-108**	0.4	0.0	1.4	
**beta_lactam__CTX-M-109**	0.2	0.0	0.8	
**beta_lactam__CTX-M-117**	0.0	0.0	0.5	
**beta_lactam__CTX-M-123**	0.3	1.9	0.3	-0.9
**beta_lactam__CTX-M-126**	0.1	0.0	0.5	
**beta_lactam__CTX-M-132**	0.1	1.2	0.3	-1.2
**beta_lactam__CTX-M-134**	0.0	0.6	0.4	-1.8
**beta_lactam__CTX-M-137**	0.0	0.3	0.4	-1.9
**beta_lactam__CTX-M-14**	0.2	0.6	0.2	-0.5
**beta_lactam__CTX-M-147**	0.0	0.6	0.4	-1.8

ARGs subtype	Hospital mean RPKM Mean	Shopping mall mean RPKM Mean	-log10 (p)	log10(FC)
**beta_lactam__CTX-M-15**	0.1	0.0	0.6	
**beta_lactam__CTX-M-160**	0.0	0.6	0.4	
**beta_lactam__CTX-M-19**	0.3	0.6	0.2	-0.3
**beta_lactam__CTX-M-21**	0.0	3.0	0.5	-2.1
**beta_lactam__CTX-M-27**	0.1	0.3	0.3	-0.5
**beta_lactam__CTX-M-3**	0.8	0.3	0.6	0.4
**beta_lactam__CTX-M-35**	0.2	0.0	0.6	
**beta_lactam__CTX-M-37**	0.1	0.0	0.5	
**beta_lactam__CTX-M-45**	0.2	1.6	0.3	-1.0
**beta_lactam__CTX-M-51**	0.0	0.3	0.4	
**beta_lactam__CTX-M-52**	0.1	0.0	0.5	
**beta_lactam__CTX-M-55**	0.4	0.6	0.1	-0.2
**beta_lactam__CTX-M-59**	0.0	0.0	0.5	
**beta_lactam__CTX-M-68**	0.3	0.0	0.9	
**beta_lactam__CTX-M-85**	0.0	0.0	0.5	
**beta_lactam__CTX-M-87**	0.0	0.0	0.5	
**beta_lactam__CTX-M-99**	0.0	0.0	0.5	
**beta_lactam__DES-1**	0.2	0.8	0.3	-0.7
**beta_lactam__DHA-1**	0.3	0.0	0.7	
**beta_lactam__DHA-12**	0.1	0.0	0.5	
**beta_lactam__DHA-20**	0.0	0.0	0.5	
**beta_lactam__DHA-22**	0.3	0.0	1.0	
**beta_lactam__DHA-7**	0.1	0.0	0.5	
**beta_lactam__DHA-9**	0.1	0.0	0.8	
**beta_lactam__Escherichia coli ampC**	3.3	2.6	0.1	0.1
**beta_lactam__ESP-1**	0.1	0.0	0.8	
**beta_lactam__FEZ-1**	0.1	0.0	0.5	
**beta_lactam__FOX-2**	0.0	0.0	0.5	
**beta_lactam__FOX-3**	0.0	0.5	0.4	-1.5
**beta_lactam__FOX-4**	0.0	0.0	0.5	

ARGs subtype	Hospital mean RPKM Mean	Shopping mall mean RPKM Mean	-log10 (p)	log10(FC)
**beta_lactam__FOX-5**	0.0	1.3	0.4	-1.9
**beta_lactam__FOX-7**	0.0	0.2	0.4	-1.2
**beta_lactam__FOX-9**	0.0	1.2	0.4	-1.9
**beta_lactam__GES-11**	0.2	0.0	1.2	
**beta_lactam__GES-12**	0.0	0.0	0.5	
**beta_lactam__GES-13**	0.6	0.0	1.7	
**beta_lactam__GES-14**	11.5	0.0	4.6	
**beta_lactam__GES-15**	18.2	0.3	6.3	1.8
**beta_lactam__GES-17**	0.2	0.0	0.9	
**beta_lactam__GES-19**	0.1	0.0	0.5	
**beta_lactam__GES-2**	0.0	0.0	0.5	
**beta_lactam__GES-20**	3.1	0.0	4.3	
**beta_lactam__GES-21**	1.3	0.0	2.6	
**beta_lactam__GES-22**	0.7	0.0	0.6	
**beta_lactam__GES-24**	1.9	0.0	3.1	
**beta_lactam__GES-26**	0.0	0.0	0.5	
**beta_lactam__GES-3**	0.2	0.0	0.9	
**beta_lactam__GES-4**	6.3	0.0	5.5	
**beta_lactam__GES-5**	4.6	0.0	6.4	
**beta_lactam__GES-6**	1.4	0.0	3.1	
**beta_lactam__GES-7**	0.3	0.0	1.5	
**beta_lactam__GIL-1**	0.1	0.0	0.9	
**beta_lactam__GOB-16**	0.2	0.0	0.6	
**beta_lactam__GOB-7**	0.0	3.1	0.4	
**beta_lactam__IDC-1**	0.0	0.0		
**beta_lactam__IDC-2**	0.0	0.0		
**beta_lactam__imiS**	0.0	0.0	0.5	
**beta_lactam__IMP-1**	24.9	1.1	7.8	1.4
**beta_lactam__IMP-10**	1.9	0.0	3.4	
**beta_lactam__IMP-11**	0.1	0.0	0.8	

ARGs subtype	Hospital mean RPKM Mean	Shopping mall mean RPKM Mean	-log10 (p)	log10(FC)
**beta_lactam__IMP-15**	0.1	0.0	0.5	
**beta_lactam__IMP-21**	0.4	0.0	1.3	
**beta_lactam__IMP-25**	0.3	0.0	1.5	
**beta_lactam__IMP-26**	0.1	0.0	0.5	
**beta_lactam__IMP-3**	1.4	0.0	1.2	
**beta_lactam__IMP-30**	0.1	0.0	0.5	
**beta_lactam__IMP-34**	0.3	0.0	1.3	
**beta_lactam__IMP-38**	0.0	0.0	0.5	
**beta_lactam__IMP-40**	2.9	0.0	3.0	
**beta_lactam__IMP-42**	15.5	1.5	4.2	1.0
**beta_lactam__IMP-5**	0.3	0.0	1.4	
**beta_lactam__IMP-52**	0.8	0.0	1.9	
**beta_lactam__IMP-55**	7.6	0.4	4.9	1.3
**beta_lactam__IMP-7**	0.0	0.0	0.5	
**beta_lactam__Klebsiella pneumoniae OmpK37**	10.0	16.7	0.4	-0.2
**beta_lactam__KPC-16**	0.3	0.0	0.8	
**beta_lactam__KPC-17**	0.2	0.0	1.0	
**beta_lactam__KPC-2**	0.0	0.0	0.5	
**beta_lactam__KPC-3**	0.0	0.0	0.5	
**beta_lactam__L1 beta-lactamase**	0.1	0.0	0.5	
**beta_lactam__LAP-1**	0.1	0.0	0.5	
**beta_lactam__LAP-2**	0.3	0.0	0.6	
**beta_lactam__Laribacter hongkongensis ampC beta-lactamase**	0.0	0.0	0.7	
**beta_lactam__LCR-1**	13.8	2.7	1.6	0.7
**beta_lactam__LEN-14**	0.0	0.0	0.5	
**beta_lactam__LEN-26**	0.0	0.0	0.5	
**beta_lactam__LEN-3**	0.0	0.0	0.5	
**beta_lactam__LEN-41**	0.0	0.9	0.4	

ARGs subtype	Hospital mean RPKM Mean	Shopping mall mean RPKM Mean	-log10 (p)	log10(FC)
**beta_lactam__LEN-6**	0.3	0.0	0.5	
**beta_lactam__LRA-1**	0.0	0.0	0.5	
**beta_lactam__LRA-17**	0.0	0.0	0.5	
**beta_lactam__LUS-1**	0.2	0.0	0.8	
**beta_lactam__MIR-14**	0.0	0.0	0.5	
**beta_lactam__MIR-23**	0.3	0.0	0.9	
**beta_lactam__MIR-6**	0.0	0.0	0.5	
**beta_lactam__MIR-9**	0.1	0.0	0.7	
**beta_lactam__MOX-1**	3.7	0.0	1.9	
**beta_lactam__MOX-2**	0.7	0.2	0.6	0.5
**beta_lactam__MOX-3**	0.6	0.0	0.7	
**beta_lactam__MOX-4**	0.3	2.1	0.4	-0.8
**beta_lactam__MOX-5**	0.0	0.0	0.5	
**beta_lactam__MOX-6**	0.0	0.0	0.5	
**beta_lactam__MOX-7**	1.0	1.2	0.1	-0.1
**beta_lactam__MOX-8**	0.0	0.0	0.5	
**beta_lactam__MOX-9**	0.0	2.1	0.4	-2.0
**beta_lactam__mreA**	0.1	0.0	0.6	
**beta_lactam__NPS-1**	14.9	0.7	6.7	1.3
**beta_lactam__OKP-A-1**	0.2	0.0	0.5	
**beta_lactam__OKP-A-11**	0.1	0.0	0.5	
**beta_lactam__OKP-A-12**	0.0	0.0	0.5	
**beta_lactam__OKP-A-15**	0.1	0.0	0.5	
**beta_lactam__OKP-A-16**	0.0	0.0	0.5	
**beta_lactam__OKP-A-2**	0.1	0.0	0.8	
**beta_lactam__OKP-A-4**	0.0	0.0	0.5	
**beta_lactam__OKP-A-8**	0.1	0.0	0.5	
**beta_lactam__OKP-B-12**	0.3	0.0	1.3	
**beta_lactam__OKP-B-13**	0.1	0.0	0.7	
**beta_lactam__OKP-B-20**	0.0	0.0	0.5	

**ARGs subtype**	Hospital mean RPKM Mean	Shopping mall mean RPKM Mean	-log10 (p)	log10(FC)
**beta_lactam__OKP-B-6**	0.1	0.0	0.6	
**beta_lactam__OKP-B-7**	0.1	0.0	0.5	
**beta_lactam__OKP-B-8**	0.1	0.0	0.6	
**beta_lactam__ORN-2**	0.5	0.0	0.6	
**beta_lactam__ORN-3**	0.5	0.0	0.8	
**beta_lactam__ORN-4**	0.2	0.0	1.0	
**beta_lactam__ORN-5**	0.3	0.3	0.0	-0.1
**beta_lactam__ORN-6**	0.1	0.0	0.5	
**beta_lactam__Other class A beta-lactamase**	21.0	5.4	0.7	0.6
**beta_lactam__Other class C beta-lactamase**	0.1	0.2	0.1	-0.2
**beta_lactam__OXA-1**	1.4	0.0	2.8	
**beta_lactam__OXA-10**	8.5	0.4	7.5	1.4
**beta_lactam__OXA-101**	0.6	0.0	2.2	
**beta_lactam__OXA-114a**	0.0	0.3	0.4	
**beta_lactam__OXA-118**	1.5	0.3	1.2	0.6
**beta_lactam__OXA-119**	3.5	0.0	5.6	
**beta_lactam__OXA-12**	0.3	4.0	0.6	-1.2
**beta_lactam__OXA-129**	0.6	0.3	0.2	0.2
**beta_lactam__OXA-13**	0.6	0.0	1.6	
**beta_lactam__OXA-140**	0.0	0.0	0.5	
**beta_lactam__OXA-142**	0.1	0.0	0.8	
**beta_lactam__OXA-145**	0.1	0.0	0.8	
**beta_lactam__OXA-147**	2.3	0.0	2.8	
**beta_lactam__OXA-15**	0.1	0.0	0.5	
**beta_lactam__OXA-164**	0.0	0.0	0.5	
**beta_lactam__OXA-17**	0.3	0.0	0.7	
**beta_lactam__OXA-18**	0.0	0.0	0.5	
**beta_lactam__OXA-182**	0.0	2.5	0.4	
**beta_lactam__OXA-183**	0.3	0.0	0.9	
**beta_lactam__OXA-19**	0.4	0.0	0.9	

**ARGs subtype**	Hospital mean RPKM Mean	Shopping mall mean RPKM Mean	-log10 (p)	log10(FC)
**beta_lactam__OXA-2**	11.4	0.0	4.2	
**beta_lactam__OXA-20**	1.5	0.0	3.1	
**beta_lactam__OXA-205**	10.3	0.0	8.1	
**beta_lactam__OXA-209**	2.0	0.0	4.2	
**beta_lactam__OXA-21**	1.1	0.0	2.8	
**beta_lactam__OXA-211**	0.0	1.0	0.4	
**beta_lactam__OXA-212**	0.0	4.2	0.7	
**beta_lactam__OXA-22**	0.1	0.0	0.8	
**beta_lactam__OXA-224**	0.1	0.0	0.5	
**beta_lactam__OXA-226**	0.8	0.0	2.7	
**beta_lactam__OXA-228**	0.1	0.0	0.5	
**beta_lactam__OXA-253**	0.0	1.0	0.4	
**beta_lactam__OXA-267**	0.0	0.0	0.5	
**beta_lactam__OXA-274**	0.3	0.0	0.5	
**beta_lactam__OXA-275**	0.0	3.1	0.5	
**beta_lactam__OXA-28**	0.1	0.0	0.9	
**beta_lactam__OXA-281**	0.0	0.0	0.5	
**beta_lactam__OXA-296**	0.0	1.0	0.4	
**beta_lactam__OXA-299**	0.0	3.9	0.4	
**beta_lactam__OXA-3**	0.4	0.0	1.4	
**beta_lactam__OXA-301**	0.1	5.1	0.4	-1.7
**beta_lactam__OXA-308**	0.1	0.0	0.5	
**beta_lactam__OXA-309**	0.0	0.7	0.4	
**beta_lactam__OXA-33**	0.1	0.0	0.5	
**beta_lactam__OXA-333**	0.0	0.3	0.4	
**beta_lactam__OXA-334**	0.0	0.7	0.4	
**beta_lactam__OXA-34**	1.9	0.0	4.1	
**beta_lactam__OXA-347**	5.0	2.5	0.4	0.3
**beta_lactam__OXA-35**	0.3	0.0	0.6	
**beta_lactam__OXA-36**	0.7	0.0	2.5	

**ARGs subtype**	Hospital mean RPKM Mean	Shopping mall mean RPKM Mean	-log10 (p)	log10(FC)
**beta_lactam__OXA-37**	0.2	0.0	1.5	
**beta_lactam__OXA-372**	0.4	0.0	0.7	
**beta_lactam__OXA-373**	0.1	2.3	0.4	-1.6
**beta_lactam__OXA-392**	0.1	0.0	0.5	
**beta_lactam__OXA-415**	0.1	0.0	0.5	
**beta_lactam__OXA-427**	1.7	12.8	0.6	-0.9
**beta_lactam__OXA-437**	0.1	0.0	0.8	
**beta_lactam__OXA-444**	0.1	0.0	0.7	
**beta_lactam__OXA-45**	0.1	0.0	0.8	
**beta_lactam__OXA-46**	0.1	0.0	0.6	
**beta_lactam__OXA-464**	0.8	0.0	2.3	
**beta_lactam__OXA-47**	0.2	0.0	0.7	
**beta_lactam__OXA-472**	0.1	0.0	0.5	
**beta_lactam__OXA-5**	0.0	0.0	0.5	
**beta_lactam__OXA-53**	0.1	0.0	0.7	
**beta_lactam__OXA-56**	0.0	0.0	0.5	
**beta_lactam__OXA-573**	0.1	0.0	0.5	
**beta_lactam__OXA-58**	0.6	0.0	1.2	
**beta_lactam__OXA-60**	0.1	0.0	0.9	
**beta_lactam__OXA-664**	0.2	0.0	0.9	
**beta_lactam__OXA-724**	0.1	6.1	0.7	-1.7
**beta_lactam__OXA-727**	0.0	2.5	0.4	-2.5
**beta_lactam__OXA-728**	0.0	2.5	0.4	-1.9
**beta_lactam__OXA-85**	0.1	0.0	0.5	
**beta_lactam__OXA-9**	0.3	0.0	1.3	
**beta_lactam__OXA-91**	0.0	1.0	0.4	
**beta_lactam__OXA-96**	0.0	0.0	0.6	
**beta_lactam__OXY-1-4**	0.2	0.0	0.6	
**beta_lactam__OXY-1-6**	0.0	0.0	0.5	
**beta_lactam__OXY-2-8**	0.1	0.0	0.5	

ARGs subtype	Hospital mean RPKM Mean	Shopping mall mean RPKM Mean	-log10 (p)	log10(FC)
**beta_lactam__OXY-3-1**	1.5	0.0	1.3	
**beta_lactam__OXY-5-1**	0.0	0.3	0.4	
**beta_lactam__PAC-1**	0.1	0.0	0.9	
**beta_lactam__PAU-1**	3.4	0.0	4.0	
**beta_lactam__PDC-1**	0.0	0.0	0.5	
**beta_lactam__PDC-7**	0.0	0.2	0.4	
**beta_lactam__PDC-91**	0.0	0.0	0.5	
**beta_lactam__PER-1**	0.0	0.0		
**beta_lactam__PLA-6**	0.1	0.0	0.5	
**beta_lactam__PME-1**	0.1	0.0	0.5	
**beta_lactam__PRC-1**	0.7	0.0	2.3	
**beta_lactam__RCP-1**	0.6	0.0	2.1	
**beta_lactam__RSD2-2**	0.1	0.4	0.3	-0.7
**beta_lactam__RUB-1**	0.4	0.0	1.6	
**beta_lactam__Sed1 beta-lactamase**	0.0	0.9	0.4	
**beta_lactam__SGM-1**	0.1	0.0	0.8	
**beta_lactam__SHV-1**	0.0	0.0	0.8	
**beta_lactam__SHV-123**	0.3	0.3	0.0	0.0
**beta_lactam__SHV-124**	0.0	0.0	0.5	
**beta_lactam__SHV-126**	0.0	0.3	0.4	
**beta_lactam__SHV-22**	0.2	0.0	1.0	
**beta_lactam__SHV-39**	0.7	1.0	0.1	-0.1
**beta_lactam__SHV-4**	0.0	1.3	0.6	
**beta_lactam__SHV-53**	0.6	0.0	1.5	
**beta_lactam__SHV-6**	0.1	0.0	0.5	
**beta_lactam__TEM-1**	2.3	0.0	2.3	
**beta_lactam__TEM-107**	0.0	0.0	0.5	
**beta_lactam__TEM-112**	0.0	0.0	0.5	
**beta_lactam__TEM-114**	0.1	0.0	0.8	
**beta_lactam__TEM-117**	4.9	0.0	2.4	

ARGs subtype	Hospital mean RPKM Mean	Shopping mall mean RPKM Mean	-log10 (p)	log10(FC)
**beta_lactam__TEM-118**	0.2	0.0	0.7	
**beta_lactam__TEM-12**	0.1	0.0	0.5	
**beta_lactam__TEM-121**	0.0	0.0	0.5	
**beta_lactam__TEM-123**	0.1	0.0	0.6	
**beta_lactam__TEM-127**	0.1	0.0	0.6	
**beta_lactam__TEM-133**	0.0	0.0	0.5	
**beta_lactam__TEM-136**	0.2	0.0	0.5	
**beta_lactam__TEM-147**	0.0	2.8	0.5	
**beta_lactam__TEM-149**	0.0	0.0	0.5	
**beta_lactam__TEM-159**	0.1	0.0	1.0	
**beta_lactam__TEM-177**	0.0	0.0	0.5	
**beta_lactam__TEM-178**	0.1	0.0	0.8	
**beta_lactam__TEM-183**	0.2	0.0	0.5	
**beta_lactam__TEM-184**	0.0	0.0	0.7	
**beta_lactam__TEM-185**	0.0	0.0	0.8	
**beta_lactam__TEM-187**	0.0	0.0	0.5	
**beta_lactam__TEM-192**	0.7	0.0	1.2	
**beta_lactam__TEM-193**	0.8	0.0	1.7	
**beta_lactam__TEM-21**	0.1	0.0	0.5	
**beta_lactam__TEM-219**	0.2	0.0	0.5	
**beta_lactam__TEM-59**	0.1	0.0	0.5	
**beta_lactam__TEM-63**	0.2	0.0	1.1	
**beta_lactam__TEM-68**	0.0	0.0	0.5	
**beta_lactam__TEM-7**	0.5	0.0	1.4	
**beta_lactam__TEM-75**	0.0	0.0	0.5	
**beta_lactam__TEM-83**	0.0	0.0	0.5	
**beta_lactam__TEM-87**	0.1	0.0	0.7	
**beta_lactam__TEM-88**	0.0	0.0	0.5	
**beta_lactam__TEM-91**	0.0	0.0	0.5	
**beta_lactam__THIN-B beta-lactamase**	0.0	0.3	0.4	-1.9

ARGs subtype	Hospital mean RPKM Mean	Shopping mall mean RPKM Mean	-log10 (p)	log10(FC)
**beta_lactam__TLA-2**	0.1	0.0	0.5	
**beta_lactam__TLA-3**	0.1	0.0	0.5	
**beta_lactam__TMB-2**	0.1	0.0	0.8	
**beta_lactam__VEB-1**	8.8	17.9	0.3	-0.3
**beta_lactam__VEB-1b**	0.0	0.0	0.5	
**beta_lactam__VEB-3**	0.3	3.2	0.5	-1.0
**beta_lactam__VEB-4**	0.0	0.0	0.5	
**beta_lactam__VEB-5**	0.1	0.0	0.5	
**beta_lactam__VEB-6**	0.1	0.0	1.2	
**beta_lactam__VEB-7**	0.1	0.0	0.9	
**beta_lactam__VEB-8**	0.1	0.0	0.5	
**beta_lactam__VEB-9**	1.0	0.0	1.2	
**beta_lactam__VIM-1**	2.2	0.6	1.2	0.6
**beta_lactam__y56**	0.0	0.3	0.4	
**bleomycin__bleomycin resistance protein**	0.3	0.0	0.5	
**bleomycin__BLMT**	0.0	0.0	0.5	
**chloramphenicol__Agrobacterium fabrum chloramphenicol acetyltransferase**	0.1	0.0	1.0	
**chloramphenicol__Bacillus clausii chloramphenicol acetyltransferase**	0.0	0.0		
**chloramphenicol__Campylobacter coli chloramphenicol acetyltransferase**	3.0	0.0	1.9	
**chloramphenicol__catA4**	0.1	0.0	0.5	
**chloramphenicol__catB10**	0.3	0.0	0.7	
**chloramphenicol__catB11**	1.6	0.9	0.3	0.3
**chloramphenicol__catB2**	0.1	0.0	0.5	
**chloramphenicol__catB3**	2.9	2.6	0.0	0.1
**chloramphenicol__catB8**	2.7	7.1	0.2	-0.4
**chloramphenicol__catD**	0.2	0.9	0.3	-0.6
**chloramphenicol__catI**	0.9	0.0	0.6	

ARGs subtype	Hospital mean RPKM Mean	Shopping mall mean RPKM Mean	-log10 (p)	log10(FC)
**chloramphenicol__catII from Escherichia coli K-12**	0.1	0.0	0.5	
**chloramphenicol__catS**	0.1	0.0	1.0	
**chloramphenicol__cmlA1**	0.0	0.0	0.5	
**chloramphenicol__cmlA5**	3.5	1.1	0.8	0.5
**chloramphenicol__cmlA6**	0.1	0.2	0.1	-0.2
**chloramphenicol__cmlA8**	0.0	0.0	0.5	
**chloramphenicol__cmlB1**	0.1	0.2	0.1	-0.2
**chloramphenicol__cmx**	0.4	0.2	0.3	0.3
**chloramphenicol__Pseudomonas aeruginos catB6**	13.2	0.0	2.1	
**chloramphenicol__Pseudomonas aeruginos catB7**	0.1	0.0	0.8	
**chloramphenicol__Salmonella enterica cmlA**	0.1	1.8	0.3	-1.1
**chloramphenicol__Vibrio anguillarum chloramphenicol acetyltransferase**	0.2	0.0	0.8	
**defensin__Bacillus subtilis mprF**	0.0	0.0	0.7	
**defensin__Brucella suis mprF**	0.0	0.1	0.3	-1.0
**florfenicol__floR**	5.3	1.7	0.8	0.5
**florfenicol__pp-flo**	0.4	0.0	1.2	
**fosfomycin__Acinetobacter baumannii AbaF**	0.3	0.2	0.1	0.1
**fosfomycin__FosA**	1.7	1.3	0.1	0.1
**fosfomycin__FosA3**	0.1	1.3	0.3	-1.0
**fosfomycin__FosA4**	0.0	1.9	0.4	
**fosfomycin__FosA5**	4.1	1.9	0.4	0.3
**fosfomycin__FosA6**	4.8	1.9	0.5	0.4
**fosfomycin__FosA7**	0.1	0.0	0.5	
**fosfomycin__FosB**	0.1	0.0	0.5	
**fosfomycin__FosX**	0.5	0.0	0.8	
**macrolide-lincosamide-streptogramin__cfr(E)**	0.5	0.0	1.0	

**ARGs subtype**	Hospital mean RPKM Mean	Shopping mall mean RPKM Mean	-log10 (p)	log10(FC)
**macrolide-lincosamide-streptogramin__cipA**	0.0	0.0	0.5	
**macrolide-lincosamide-streptogramin__clcD**	0.1	0.0	0.5	
**macrolide-lincosamide-streptogramin__ere(A)**	28.4	3.9	3.3	0.9
**macrolide-lincosamide-streptogramin__ere(B)**	1.9	1.9	0.0	0.0
**macrolide-lincosamide-streptogramin__ere(D)**	5.1	3.7	0.3	0.1
**macrolide-lincosamide-streptogramin__ereA2**	0.0	0.0	0.6	
**macrolide-lincosamide-streptogramin__erm(31)**	0.0	0.0	0.5	
**macrolide-lincosamide-streptogramin__erm(35)**	1.8	0.3	1.5	0.7
**macrolide-lincosamide-streptogramin__erm(36)**	0.1	0.0	0.6	
**macrolide-lincosamide-streptogramin__erm(39)**	0.0	0.0	0.7	
**macrolide-lincosamide-streptogramin__erm(40)**	0.0	0.0	0.7	
**macrolide-lincosamide-streptogramin__erm(42)**	1.2	0.0	1.5	
**macrolide-lincosamide-streptogramin__erm(47)**	0.0	0.0	0.5	
**macrolide-lincosamide-streptogramin__erm(50)**	0.0	0.7	0.4	
**macrolide-lincosamide-streptogramin__Erm(52)**	0.2	0.0	1.0	
**macrolide-lincosamide-streptogramin__erm(A)**	0.8	0.0	1.7	
**macrolide-lincosamide-streptogramin__erm(B)**	23.3	66.2	0.5	-0.5

**ARGs subtype**	Hospital mean RPKM Mean	Shopping mall mean RPKM Mean	-log10 (p)	log10(FC)
**macrolide-lincosamide-streptogramin__erm(C)**	0.0	0.0	0.5	
**macrolide-lincosamide-streptogramin__erm(F)**	45.7	27.0	0.7	0.2
**macrolide-lincosamide-streptogramin__erm(G)**	23.5	12.4	0.7	0.3
**macrolide-lincosamide-streptogramin__erm(T)**	0.2	0.0	1.0	
**macrolide-lincosamide-streptogramin__erm(X)**	3.5	2.8	0.1	0.1
**macrolide-lincosamide-streptogramin__linG**	0.7	0.0	2.0	
**macrolide-lincosamide-streptogramin__LlmA 23S ribosomal RNA methyltransferase**	1.4	0.6	0.4	0.4
**macrolide-lincosamide-streptogramin__lmrB**	0.0	0.0	0.5	
**macrolide-lincosamide-streptogramin__lmrC**	0.0	0.0	0.5	
**macrolide-lincosamide-streptogramin__lmrD**	0.1	10.9	0.8	-2.1
**macrolide-lincosamide-streptogramin__lnu(B)**	0.0	0.0	0.5	
**macrolide-lincosamide-streptogramin__lnu(C)**	4.5	9.6	0.5	-0.3
**macrolide-lincosamide-streptogramin__lnu(D)**	1.2	0.6	0.4	0.4
**macrolide-lincosamide-streptogramin__lnu(F)**	0.3	0.0	1.2	
**macrolide-lincosamide-streptogramin__lnu(H)**	2.1	2.7	0.1	-0.1
**macrolide-lincosamide-streptogramin__lnu(P)**	0.0	0.0	0.5	
**macrolide-lincosamide-streptogramin__LpeB**	0.0	0.0	0.5	
**macrolide-lincosamide-streptogramin__lsa(A)**	0.0	1.8	0.5	-1.6
**macrolide-lincosamide-streptogramin__lsa(B)**	2.3	0.7	1.3	0.5

ARGs subtype	Hospital mean RPKM Mean	Shopping mall mean RPKM Mean	-log10 (p)	log10(FC)
**macrolide-lincosamide-streptogramin__lsa(E)**	1.3	0.3	0.3	0.6
**macrolide-lincosamide-streptogramin__macA**	5.0	10.1	0.4	-0.3
**macrolide-lincosamide-streptogramin__macB**	7.2	14.5	0.5	-0.3
**macrolide-lincosamide-streptogramin__mef(B)**	1.3	2.1	0.2	-0.2
**macrolide-lincosamide-streptogramin__mef(C)**	3.3	2.4	0.2	0.1
**macrolide-lincosamide-streptogramin__mef(E)**	0.1	0.0	0.7	
**macrolide-lincosamide-streptogramin__mef(En2)**	10.6	5.1	1.1	0.3
**macrolide-lincosamide-streptogramin__mel**	17.8	11.5	0.5	0.2
**macrolide-lincosamide-streptogramin__mph(A)**	5.1	8.5	0.3	-0.2
**macrolide-lincosamide-streptogramin__mph(B)**	1.7	1.1	0.1	0.2
**macrolide-lincosamide-streptogramin__mph(F)**	0.1	1.2	0.4	-1.4
**macrolide-lincosamide-streptogramin__mph(G)**	2.0	0.6	0.8	0.5
**macrolide-lincosamide-streptogramin__mph(K)**	0.0	0.0	0.5	
**macrolide-lincosamide-streptogramin__msr(C)**	0.3	0.0	1.3	
**macrolide-lincosamide-streptogramin__optrA**	0.1	0.1	0.2	-0.3
**macrolide-lincosamide-streptogramin__poxtA**	0.2	0.0	1.3	
**macrolide-lincosamide-streptogramin__RlmA(II)**	0.3	0.0	0.9	
**macrolide-lincosamide-streptogramin__vat(A)**	0.2	0.0	0.5	

ARGs subtype	Hospital mean RPKM Mean	Shopping mall mean RPKM Mean	-log10 (p)	log10(FC)
**macrolide-lincosamide-streptogramin__vat(B)**	2.0	7.0	0.3	-0.5
**macrolide-lincosamide-streptogramin__vat(D)**	0.0	0.4	0.4	
**macrolide-lincosamide-streptogramin__vat(F)**	2.1	3.2	0.2	-0.2
**macrolide-lincosamide-streptogramin__vat(H)**	0.0	0.0	0.5	
**macrolide-lincosamide-streptogramin__VatI**	0.0	0.0	0.5	
**macrolide-lincosamide-streptogramin__vmlR**	0.0	0.0	0.5	
**multidrug__abeM**	2.2	18.3	0.7	-0.9
**multidrug__abeS**	0.2	0.0	0.6	
**multidrug__Acinetobacter baumannii AmvA**	0.2	0.0	0.7	
**multidrug__acrE**	3.1	2.2	0.8	0.1
**multidrug__acrF**	7.9	10.9	0.3	-0.1
**multidrug__adeF**	1.5	0.6	1.2	0.4
**multidrug__adeH**	0.1	0.1	0.0	-0.1
**multidrug__adeI**	0.6	8.4	0.7	-1.1
**multidrug__amrB**	0.9	1.2	0.3	-0.1
**multidrug__blt**	0.0	1.7	0.4	-2.8
**multidrug__bmr**	0.0	0.0	0.5	
**multidrug__bpeF**	0.3	0.0	2.8	0.9
**multidrug__ceoB**	0.8	0.8	0.0	0.0
**multidrug__cmeC**	0.0	0.0	0.5	
**multidrug__efmA**	0.5	0.0	1.5	
**multidrug__efpA**	1.4	0.0	3.7	
**multidrug__efrA**	0.8	0.9	0.1	-0.1
**multidrug__efrB**	1.6	1.1	0.2	0.2
**multidrug__emeA**	1.5	0.2	0.8	0.8
**multidrug__emrA**	6.9	11.5	0.5	-0.2
**multidrug__emrB**	7.4	19.1	1.0	-0.4

ARGs subtype	Hospital mean RPKM Mean	Shopping mall mean RPKM Mean	-log10 (p)	log10(FC)
**multidrug__emrD**	22.1	21.5	0.0	0.0
**multidrug__emrK**	0.9	2.1	0.6	-0.4
**multidrug__emrY**	0.9	0.8	0.0	0.0
**multidrug__Enterobacter cloacae acrA**	0.4	0.2	0.7	0.5
**multidrug__Escherichia coli acrA**	1.3	1.1	0.0	0.1
**multidrug__Escherichia coli emrE**	3.5	16.3	0.7	-0.7
**multidrug__Escherichia coli mdfA**	14.4	20.6	0.9	-0.2
**multidrug__fexB**	0.0	0.0	0.5	
**multidrug__Klebsiella pneumoniae acrA**	2.7	2.5	0.1	0.0
**multidrug__Klebsiella pneumoniae KpnG**	0.1	0.0	0.6	
**multidrug__lmrP**	0.2	2.8	0.7	-1.1
**multidrug__mdsA**	0.1	0.0	0.9	
**multidrug__mdsB**	0.7	0.1	2.4	1.1
**multidrug__mdsC**	0.0	0.0	0.5	
**multidrug__mdtE**	5.3	6.0	0.1	-0.1
**multidrug__mdtF**	2.8	2.3	0.2	0.1
**multidrug__mdtH**	14.0	16.1	0.2	-0.1
**multidrug__mdtK**	21.4	41.4	0.6	-0.3
**multidrug__mdtL**	13.2	12.7	0.0	0.0
**multidrug__mdtM**	9.2	7.1	0.2	0.1
**multidrug__mdtN**	1.7	0.9	0.3	0.3
**multidrug__mdtO**	1.8	1.6	0.1	0.0
**multidrug__mdtP**	2.1	4.4	0.4	-0.3
**multidrug__mepA**	0.1	0.0	0.5	
**multidrug__MexA**	0.6	0.8	0.1	-0.1
**multidrug__MexB**	8.2	11.8	2.0	-0.2
**multidrug__MexC**	1.4	1.0	0.3	0.1
**multidrug__MexD**	3.2	1.0	2.0	0.5
**multidrug__MexE**	3.6	12.1	0.7	-0.5
**multidrug__MexF**	2.3	1.5	0.8	0.2

ARGs subtype	Hospital mean RPKM Mean	Shopping mall mean RPKM Mean	-log10 (p)	log10(FC)
**multidrug__mexG**	0.0	0.0	0.5	
**multidrug__mexH**	0.0	0.0	0.5	
**multidrug__mexJ**	0.2	0.5	0.5	-0.4
**multidrug__mexK**	2.9	7.4	0.7	-0.4
**multidrug__mexM**	0.0	0.0	0.5	
**multidrug__mexN**	0.2	0.3	0.1	-0.1
**multidrug__mexP**	0.2	0.0	0.6	
**multidrug__mexQ**	0.2	0.0	1.8	0.9
**multidrug__mexV**	0.0	0.0	0.5	
**multidrug__mexW**	1.4	2.0	0.2	-0.1
**multidrug__msbA**	18.3	32.3	0.7	-0.2
**multidrug__mtrC**	0.0	0.0	0.5	
**multidrug__mtrD**	0.6	1.9	0.5	-0.5
**multidrug__mtrE**	0.0	0.7	0.6	-1.1
**multidrug__MuxA**	0.1	0.0	1.3	
**multidrug__MuxB**	5.2	5.2	0.0	0.0
**multidrug__MuxC**	1.2	0.8	1.5	0.2
**multidrug__opcM**	0.0	0.0	0.7	
**multidrug__OpmB**	0.2	0.0	1.2	
**multidrug__opmD**	0.0	0.1	0.3	-0.7
**multidrug__opmE**	0.1	0.0	0.5	
**multidrug__OpmH**	0.9	0.6	0.2	0.2
**multidrug__oprA**	0.3	0.5	0.2	-0.3
**multidrug__oprC**	0.2	0.0	4.7	1.1
**multidrug__OprJ**	0.7	0.3	0.5	0.4
**multidrug__OprM**	2.2	6.3	1.2	-0.5
**multidrug__OprN**	0.3	0.3	0.0	0.0
**multidrug__OprZ**	0.0	0.0	0.5	
**multidrug__oqxA**	2.4	3.8	0.3	-0.2
**multidrug__oqxB**	3.4	5.2	0.3	-0.2

ARGs subtype	Hospital mean RPKM Mean	Shopping mall mean RPKM Mean	-log10 (p)	log10(FC)
**multidrug__Other_major_facilitator_superfamily_transporter**	1.1	0.2	1.5	0.7
**multidrug__ParS**	0.0	0.0	0.5	
**multidrug__patA**	0.1	1.3	0.4	-1.3
**multidrug__patB**	0.3	0.2	0.3	0.3
**multidrug__PmpM**	0.6	0.2	0.8	0.5
**multidrug__pmrA**	0.1	0.0	0.8	
**multidrug__qacE**	19.1	14.3	0.1	0.1
**multidrug__qacEdelta1**	57.8	13.9	2.6	0.6
**multidrug__qacH**	35.0	80.5	0.7	-0.4
**multidrug__RanA**	19.3	11.6	0.9	0.2
**multidrug__RanB**	13.0	11.5	0.1	0.1
**multidrug__sdeY**	3.1	4.3	0.8	-0.1
**multidrug__smeD**	0.4	0.0	1.7	
**multidrug__smeE**	2.0	3.7	0.8	-0.3
**multidrug__smeF**	0.0	0.2	0.3	-0.7
**multidrug__tap**	0.5	0.0	1.8	
**multidrug__tolC**	7.4	10.8	0.3	-0.2
**multidrug__ykkD**	0.0	0.0	0.5	
**mupirocin__Bifidobacteria intrinsic ileS conferring resistance to mupirocin**	9.7	12.8	0.3	-0.1
**mupirocin__mupA**	0.1	0.2	0.2	-0.4
**mupirocin__mupB**	0.3	0.0	2.4	
**novobiocin__novA**	0.8	2.4	0.3	-0.5
**other_peptide_antibiotics__ArnT**	13.8	13.8	0.0	0.0
**other_peptide_antibiotics__microcin efflux pumu gene yojI**	12.2	17.3	0.5	-0.2
**other_peptide_antibiotics__thiostrepton resistance gene tsnR**	0.0	0.0	0.5	
**pleuromutilin_tiamulin__TaeA**	1.4	1.9	0.2	-0.1

ARGs subtype	Hospital mean RPKM Mean	Shopping mall mean RPKM Mean	-log10 (p)	log10(FC)
**polymyxin__arnA**	20.8	21.8	0.1	0.0
**polymyxin__eptA**	9.5	11.5	0.1	-0.1
**polymyxin__ICR-Mo**	0.3	0.6	0.3	-0.4
**polymyxin__mcr-10.1**	0.3	1.5	0.3	-0.6
**polymyxin__mcr-3.1**	0.1	0.5	0.3	-0.9
**polymyxin__mcr-3.10**	0.0	0.0	0.5	
**polymyxin__mcr-3.11**	0.0	0.0	0.5	
**polymyxin__mcr-3.12**	0.0	0.3	0.4	
**polymyxin__mcr-3.19**	0.0	0.2	0.3	-0.7
**polymyxin__mcr-3.2**	0.0	0.0	0.5	
**polymyxin__mcr-3.20**	0.0	0.0	0.5	
**polymyxin__mcr-3.3**	0.1	0.3	0.2	-0.4
**polymyxin__mcr-3.4**	0.0	0.2	0.4	-1.5
**polymyxin__mcr-3.5**	0.0	0.2	0.3	-1.0
**polymyxin__mcr-3.6**	0.0	0.0	0.5	
**polymyxin__mcr-3.7**	0.0	1.6	0.5	
**polymyxin__mcr-3.8**	0.1	0.0	1.0	
**polymyxin__mcr-3.9**	0.1	0.2	0.1	-0.2
**polymyxin__mcr-4.3**	0.0	0.0	0.5	
**polymyxin__mcr-5.1**	0.2	0.0	0.9	
**polymyxin__mcr-5.2**	0.0	0.0	0.7	
**polymyxin__mcr-7.1**	0.1	1.5	0.3	-1.2
**polymyxin__mcr-9.1**	0.7	1.0	0.2	-0.2
**polymyxin__pmrF**	14.2	25.1	0.5	-0.2
**polymyxin__rosA**	5.0	10.1	0.6	-0.3
**polymyxin__rosB**	8.2	10.0	0.3	-0.1
**polymyxin__ugd**	44.8	91.5	0.6	-0.3
**quinolone__Acinetobacter baumannii AbaQ**	0.2	0.4	0.2	-0.3
**quinolone__lfrA**	0.6	0.4	0.2	0.2
**quinolone__mfpA**	0.3	0.0	0.6	

ARGs subtype	Hospital mean RPKM Mean	Shopping mall mean RPKM Mean	-log10 (p)	log10(FC)
**quinolone__norB**	0.0	0.0	0.8	
**quinolone__qepA**	0.0	0.0	0.8	
**quinolone__QnrA1**	0.2	1.7	0.3	-0.9
**quinolone__QnrA2**	0.0	0.4	0.4	
**quinolone__QnrA3**	0.0	0.0	0.5	
**quinolone__QnrB11**	0.0	0.0	0.5	
**quinolone__QnrB27**	0.1	0.0	0.6	
**quinolone__QnrB3**	0.0	0.0	0.5	
**quinolone__QnrB30**	0.5	0.0	0.7	
**quinolone__QnrB35**	0.0	3.3	0.4	
**quinolone__QnrB5**	0.1	0.0	0.6	
**quinolone__QnrB50**	0.2	0.0	0.8	
**quinolone__QnrB54**	0.2	0.0	0.9	
**quinolone__QnrB55**	0.1	0.0	0.5	
**quinolone__QnrB65**	0.1	0.0	0.5	
**quinolone__QnrB67**	0.2	0.0	0.9	
**quinolone__QnrB7**	0.1	0.0	0.5	
**quinolone__QnrB74**	0.0	0.0	0.5	
**quinolone__QnrB8**	0.3	0.0	0.5	
**quinolone__QnrD1**	0.3	0.4	0.1	-0.1
**quinolone__QnrD2**	0.0	0.0	0.5	
**quinolone__QnrS10**	0.3	0.0	0.9	
**quinolone__QnrS11**	0.0	0.0	0.5	
**quinolone__QnrS15**	0.3	0.0	1.1	
**quinolone__QnrS2**	4.3	44.1	0.6	-1.0
**quinolone__QnrS3**	0.1	0.0	0.5	
**quinolone__QnrS5**	1.2	1.2	0.0	0.0
**quinolone__QnrS6**	2.8	34.2	0.4	-1.1
**quinolone__QnrS7**	0.0	0.0	0.5	
**quinolone__QnrS8**	0.1	1.2	0.3	-1.0

**ARGs subtype**	Hospital mean RPKM Mean	Shopping mall mean RPKM Mean	-log10 (p)	log10(FC)
**quinolone__QnrS9**	0.1	0.0	0.5	
**quinolone__QnrVC1**	0.2	0.0	0.5	
**quinolone__QnrVC4**	0.0	0.0	0.5	
**quinolone__QnrVC5**	0.1	0.0	0.5	
**quinolone__QnrVC6**	0.0	0.0	0.5	
**rifamycin__arr-1**	0.5	0.0	0.9	
**rifamycin__arr-2**	0.9	2.4	0.2	-0.4
**rifamycin__arr-3**	0.6	0.0	1.0	
**rifamycin__arr-8**	0.2	0.0	0.5	
**rifamycin__iri**	0.3	0.0	1.1	
**rifamycin__RbpA**	0.7	0.0	1.8	
**rifamycin__rphA**	0.2	0.0	1.2	
**rifamycin__rphB**	0.2	0.3	0.1	-0.2
**streptothricin__SAT-2**	0.2	0.0	1.1	
**streptothricin__SAT-4**	3.8	2.0	0.3	0.3
**sulfonamide__sul1**	155.7	64.4	1.3	0.4
**sulfonamide__sul2**	15.0	6.0	0.7	0.4
**sulfonamide__sul3**	0.0	0.0	0.5	
**sulfonamide__sul4**	0.1	0.0	1.4	
**tetracycline__otr(A)**	0.0	0.1	0.3	-1.0
**tetracycline__otr(C)**	0.0	0.0	0.5	
**tetracycline__tet(30)**	0.2	0.0	0.7	
**tetracycline__tet(31)**	0.0	0.0		
**tetracycline__tet(32)**	12.8	3.5	1.8	0.6
**tetracycline__tet(33)**	0.3	6.2	0.4	-1.3
**tetracycline__tet(34)**	3.2	2.3	0.2	0.1
**tetracycline__tet(35)**	0.1	0.2	0.3	-0.5
**tetracycline__tet(36)**	3.4	6.4	0.2	-0.3
**tetracycline__tet(37)**	6.1	13.8	0.2	-0.4
**tetracycline__tet(39)**	3.0	47.8	0.5	-1.2

**ARGs subtype**	Hospital mean RPKM Mean	Shopping mall mean RPKM Mean	-log10 (p)	log10(FC)
**tetracycline__tet(40)**	9.2	1.8	1.7	0.7
**tetracycline__tet(42)**	0.3	0.0	0.8	
**tetracycline__tet(43)**	0.7	0.0	1.4	
**tetracycline__tet(44)**	0.6	0.1	1.0	0.6
**tetracycline__tet(51)**	0.0	0.0	0.5	
**tetracycline__tet(57)**	0.0	0.0	0.5	
**tetracycline__tet(59)**	0.0	0.0	0.7	
**tetracycline__tet(A)**	5.3	4.3	0.1	0.1
**tetracycline__tet(B)**	0.2	0.0	1.0	
**tetracycline__tet(C)**	10.1	5.6	1.7	0.3
**tetracycline__tet(D)**	0.1	2.1	0.4	-1.6
**tetracycline__tet(E)**	0.3	1.8	0.3	-0.7
**tetracycline__tet(G)**	3.5	1.2	0.8	0.5
**tetracycline__tet(L)**	0.7	0.0	1.6	
**tetracycline__tet(M)**	8.3	1.9	2.1	0.6
**tetracycline__tet(O)**	19.9	10.9	0.6	0.3
**tetracycline__tet(O/W)**	2.2	2.2	0.0	0.0
**tetracycline__tet(Q)**	106.1	90.9	0.1	0.1
**tetracycline__tet(S)**	0.0	1.4	0.4	-2.1
**tetracycline__tet(T)**	0.2	0.1	0.0	0.1
**tetracycline__tet(U)**	0.9	0.0	1.2	
**tetracycline__tet(V)**	0.2	0.0	1.1	
**tetracycline__tet(W)**	26.8	20.5	0.2	0.1
**tetracycline__tet(W/N/W)**	1.0	2.5	0.9	-0.4
**tetracycline__tet(X)**	0.6	0.0	2.9	
**tetracycline__tet(X3)**	1.1	0.0	2.8	
**tetracycline__tet(X4)**	0.1	0.0	0.8	
**tetracycline__tet(X5)**	0.2	0.5	0.2	-0.4
**tetracycline__tet(Y)**	0.3	0.0	1.2	
**tetracycline__tetA**	0.1	0.0	0.5	

ARGs subtype	Hospital mean RPKM Mean	Shopping mall mean RPKM Mean	-log10 (p)	log10(FC)
**tetracycline__tetA(46)**	0.1	0.2	0.1	-0.2
**tetracycline__tetA(48)**	1.1	4.9	0.4	-0.7
**tetracycline__tetA(60)**	0.0	0.0	0.6	
**tetracycline__tetA(P)**	0.1	0.0	0.8	
**tetracycline__tetB(46)**	0.2	0.1	0.6	0.4
**tetracycline__tetB(48)**	0.3	0.0	1.2	
**tetracycline__tetB(60)**	0.1	0.0	1.1	
**tetracycline__tetB(P)**	0.1	0.1	0.0	-0.1
**tetracycline__tetX1**	1.7	0.3	1.0	0.8
**tetracycline__tetX2**	10.8	8.1	0.2	0.1
**tetracycline__tetX6**	0.5	0.2	0.4	0.3
**trimethoprim__dfrA1**	0.9	0.0	0.5	
**trimethoprim__dfrA12**	0.6	0.0	1.1	
**trimethoprim__dfrA14**	0.7	1.2	0.1	-0.2
**trimethoprim__dfrA15**	0.2	0.0	0.5	
**trimethoprim__dfrA16**	0.2	0.0	0.8	
**trimethoprim__dfrA17**	1.4	0.6	0.4	0.4
**trimethoprim__dfrA22**	0.5	0.0	1.2	
**trimethoprim__dfrA25**	0.0	0.0	0.5	
**trimethoprim__dfrA27**	0.4	0.0	0.6	
**trimethoprim__dfrA29**	0.1	0.0	0.5	
**trimethoprim__dfrA3**	1.6	6.0	0.4	-0.6
**trimethoprim__DfrA36**	0.1	0.0	0.5	
**trimethoprim__dfrA5**	0.5	0.0	0.7	
**trimethoprim__dfrA7**	0.1	0.0	0.5	
**trimethoprim__dfrE**	0.3	0.0	0.5	
**trimethoprim__dfrF**	2.6	1.1	0.5	0.4
**vancomycin__vanA**	0.0	0.0	0.5	
**vancomycin__vanB**	1.5	0.0	3.0	
**vancomycin__vanC**	0.0	0.0	0.5	

**ARGs subtype**	Hospital mean RPKM Mean	Shopping mall mean RPKM Mean	-log10 (p)	log10(FC)
**vancomycin__vanD**	0.1	0.0	0.9	
**vancomycin__vanF**	0.2	0.0	0.7	
**vancomycin__vanG**	0.3	2.3	0.4	-0.9
**vancomycin__vanHA**	0.1	0.0	0.8	
**vancomycin__vanHB**	0.7	0.0	2.6	
**vancomycin__vanHD**	0.1	0.0	0.8	
**vancomycin__vanHF**	0.1	0.0	0.6	
**vancomycin__vanHO**	1.2	0.0	3.0	
**vancomycin__vanI**	0.1	0.0	1.1	
**vancomycin__vanO**	2.6	0.0	3.8	
**vancomycin__vanTG**	0.0	0.1	0.4	-1.9
**vancomycin__vanUG**	0.1	0.0	0.5	
**vancomycin__vanXA**	0.1	0.0	1.0	
**vancomycin__vanXB**	1.1	0.0	1.9	
**vancomycin__vanXD**	0.6	0.0	1.0	
**vancomycin__vanXF**	0.3	0.0	1.0	
**vancomycin__vanXI**	0.0	0.0	0.5	
**vancomycin__vanXO**	3.6	0.0	2.5	
**vancomycin__vanYB**	0.6	0.0	1.8	
**vancomycin__vanYD**	0.1	0.0	0.9	
**vancomycin__vanYG1**	0.2	0.0	0.7	

Abbreviations: FC; fold change, RPKM; reads per kilobase per million.

Supplemental Table S2. Sampling date of wastewater samples.

	Date
Hospital sample 1	2019/12/23
Hospital sample 2	2020/01/27
Hospital sample 3	2020/02/28
Hospital sample 4	2020/03/30
Hospital sample 5	2020/04/30
Hospital sample 6	2020/05/26
Hospital sample 7	2020/06/29
Hospital sample 8	2020/07/27
Hospital sample 9	2020/08/26
Hospital sample 10	2020/09/28
Hospital sample 11	2020/10/27
Hospital sample 12	2020/11/30
Hospital sample 13	2020/12/23
Hospital sample 14	2021/01/26
Hospital sample 15	2021/02/24
Hospital sample 16	2021/03/24
Hospital sample 17	2021/04/26
Hospital sample 18	2021/05/31
Hospital sample 19	2021/06/29
Hospital sample 20	2021/07/27
Hospital sample 21	2021/08/30
Hospital sample 22	2021/09/21
Hospital sample 23	2021/10/26
Hospital sample 24	2021/11/29
Hospital sample 25	2021/12/21
Hospital sample 26	2022/01/26
Hospital sample 27	2022/02/28
Hospital sample 28	2022/03/28
Hospital sample 29	2022/04/25
Hospital sample 30	2022/05/25
Hospital sample 31	2022/06/27

	Date
Hospital sample 32	2022/07/25
Hospital sample 33	2022/08/29
Hospital sample 34	2022/09/28
Hospital sample 35	2022/10/31
Hospital sample 36	2022/11/29
Hospital sample 37	2022/12/26
Hospital sample 38	2023/01/30
Hospital sample 39	2023/02/28
Hospital sample 40	2023/03/27
Hospital sample 41	2023/04/24
Hospital sample 42	2023/05/29
Hospital sample 43	2023/06/26
Hospital sample 44	2023/07/25
Hospital sample 45	2023/08/28
Hospital sample 46	2023/09/25
Shopping mall sample 1	2020/07/27
Shopping mall sample 2	2021/03/01
Shopping mall sample 3	2020/07/27

Supplemental Table S3. Generalized liner model to evaluate association between mean RPKM by antimicrobial class and monthly trend.

	Coefficient [95% CI]	p-value
Aminoglycoside	-0.0001 [-0.0021, 0.0018]	0.903
Bacitracin	-0.0012 [-0.0168, 0.0144]	0.882
B-lactam	0.0000 [-0.0005, 0.0005]	0.998
Chloramphenicol	-0.0010 [-0.0016, -0.0004]	0.002
Florfenicol	0.0006 [-0.0022, 0.0035]	0.653
Fosfomycin	-0.0008 [-0.0022, 0.0006]	0.262
Macrolide, lincosamide, streptogramin	-0.0003 [-0.0178, 0.0104]	0.605
Muti-drugs	-0.0007 [-0.0026, 0.0012]	0.464
Mupirocin	-0.0019 [-0.0048, 0.0011]	0.210
Other peptide antimicrobials	-0.0064 [-0.0139, 0.00102]	0.091
Polymyxin	-0.0013 [-0.0034, 0.0008]	0.224
Quinolone	0.0004 [0.0001, 0.0007]	0.017
Rifamycin	-0.0002 [-0.0007, 0.0002]	0.321
Tetracycline	0.0004 [-0.0024, 0.0031]	0.793
Trimethoprim	0.0000 [-0.0005, 0.0005]	0.880
Vancomycin	0.0000 [-0.0003, 0.0003]	0.784

Positive coefficient represents an increasing trend over time.

The generalized linear model was implemented using gamma distribution and identity function with replacing zero values with 0.01.

The mean RPKM was calculated by the average of RPKM of ARGs detected either by hospital or community samples.

Supplemental Table S4. Temporal variations in the ratio of clarithromycin and levofloxacin in wastewater from a hospital compared to a shopping mall.

Sampling date		Ratio of the hospital to the shopping mall		Log_{10} value	
		Clarithromycin	Levofloxacin	Clarithromycin	Levofloxacin
2020	2020/5/26	0.3	6.6	-0.525	0.817
	2020/8/26	8.5	7.9	0.929	0.899
	2020/11/30	41	63	1.615	1.801
2021	2021/1/26	7.9	137	0.896	2.137
	2021/2/24	57	41	1.756	1.612
	2021/4/26	0.6	109	-0.240	2.038
	2021/5/31	1.0	225	0.007	2.352
	2021/6/29	2.4	47	0.373	1.673
	2021/7/27	8.9	43	0.951	1.633
	2021/8/30	N.A.	16	N.A.	1.200
	2021/9/21	1.5	52	0.179	1.714
	2021/10/26	N.A.	17	N.A.	1.222
	2021/11/29	2.4	15	0.371	1.186
	2021/12/21	76	535	1.880	2.728
2022	2022/2/28	1.5	299	0.182	2.475
	2022/3/28	30	61	1.473	1.786
	2022/4/25	N.A.	198	N.A.	2.297
	2022/5/25	N.A.	20	N.A.	1.296
	2022/6/27	11	360	1.040	2.556
	2022/7/25	1.0	40	-0.014	1.606
	2022/8/29	1.0	192	0.001	2.282
	2022/9/28	1.1	98	0.029	1.992
	2022/10/31	4.9	680	0.691	2.832
	2022/11/29	14	855	1.137	2.932
	2022/12/26	2.9	21	0.470	1.315
2023	2023/1/30	2.8	22	0.446	1.344
	2023/2/28	N.A.	79	N.A.	1.895
	2023/3/27	1.7	60	0.238	1.775
	2023/4/24	1.0	534	0.014	2.727
	2023/5/29	10	290	0.980	2.462
Mean		12	171	0.595	1.886
SD		19	219	0.648	0.587

NA; Not applicable, SD; standard deviation.

Supplemental Table S5. All raw read sequence files in the DRA/SRA database.

Sample Name	BioSample	Run
N-20191223-SewageEffluent-DNAseq	SAMD00909688	DRR680724
N-20200127-SewageEffluent-DNAseq	SAMD00909689	DRR680725
N-20200228-SewageEffluent-DNAseq	SAMD00909690	DRR680726
N-20200330-SewageEffluent-DNAseq	SAMD00909691	DRR680727
N-20200430-SewageEffluent-DNAseq	SAMD00909692	DRR680728
N-20200526-SewageEffluent-DNAseq	SAMD00909693	DRR680729
N-20200629-SewageEffluent-DNAseq	SAMD00909694	DRR680730
N-20200727-SewageEffluent-DNAseq	SAMD00909695	DRR680731
N-20200826-SewageEffluent-DNAseq	SAMD00909696	DRR680732
N-20200928-SewageEffluent-DNAseq	SAMD00909697	DRR680733
N-20201027-SewageEffluent-DNAseq	SAMD00909698	DRR680734
N-20201130-SewageEffluent-DNAseq	SAMD00909699	DRR680735
N-20201223-SewageEffluent-DNAseq	SAMD00909700	DRR680736
N-20210126-SewageEffluent-DNAseq	SAMD00909701	DRR680737
N-20210224-SewageEffluent-DNAseq	SAMD00909702	DRR680738
N-20210324-SewageEffluent-DNAseq	SAMD00909703	DRR680739

Sample Name	BioSample	Run
N-20210426-SewageEffluent-DNAseq	SAMD00909704	DRR680740
N-20210531-SewageEffluent-DNAseq	SAMD00909705	DRR680741
N-20210629-SewageEffluent-DNAseq	SAMD00909706	DRR680742
N-20210727-SewageEffluent-DNAseq	SAMD00909707	DRR680743
N-20210830-SewageEffluent-DNAseq	SAMD00909708	DRR680744
N-20210921-SewageEffluent-DNAseq	SAMD00909709	DRR680745
N-20211026-SewageEffluent-DNAseq	SAMD00909710	DRR680746
N-20211129-SewageEffluent-DNAseq	SAMD00909711	DRR680747
N-20211221-SewageEffluent-DNAseq	SAMD00909712	DRR680748
N-20220126-SewageEffluent-DNAseq	SAMD00909713	DRR680749
N-20220228-SewageEffluent-DNAseq	SAMD00909714	DRR680750
N-20220328-SewageEffluent-DNAseq	SAMD00909715	DRR680751
N-20220425-SewageEffluent-DNAseq	SAMD00909716	DRR680752
N-20220525-SewageEffluent-DNAseq	SAMD00909717	DRR680753
N-20220627-SewageEffluent-DNAseq	SAMD00909718	DRR680754
N-20220725-SewageEffluent-DNAseq	SAMD00909719	DRR680755
N-20220829-SewageEffluent-DNAseq	SAMD00909720	DRR680756

Sample Name	BioSample	Run
N-20220928-SewageEffluent-DNAseq	SAMD00909721	DRR680757
N-20221031-SewageEffluent-DNAseq	SAMD00909722	DRR680758
N-20221129-SewageEffluent-DNAseq	SAMD00909723	DRR680759
N-20221226-SewageEffluent-DNAseq	SAMD00909724	DRR680760
N-20230130-SewageEffluent-DNAseq	SAMD00909725	DRR680761
N-20230228-SewageEffluent-DNAseq	SAMD00909726	DRR680762
N-20230327-SewageEffluent-DNAseq	SAMD00909727	DRR680763
N-20230424-SewageEffluent-DNAseq	SAMD00909728	DRR680764
N-20230529-SewageEffluent-DNAseq	SAMD00909729	DRR680765
N-20230626-SewageEffluent-DNAseq	SAMD00909730	DRR680766
N-20230725-SewageEffluent-DNAseq	SAMD00909731	DRR680767
N-20230828-SewageEffluent-DNAseq	SAMD00909732	DRR680768
N-20230925-SewageEffluent-DNAseq	SAMD00909733	DRR680769
N-F-20250204	SAMD00909734	DRR680770
N-F-20250218	SAMD00909735	DRR680771
N-F-20250225	SAMD00909736	DRR680772
SWE-MallH-C-20-0727	SAMD00909737	DRR680773
SWE-MallH-C-21-0301	SAMD00909738	DRR680774
SWE-MallH-E-20-0727	SAMD00909739	DRR680775
